# Prodrug polymeric micelles integrating cancer-associated fibroblasts deactivation and synergistic chemotherapy for gastric cancer

**DOI:** 10.1186/s12951-021-01127-5

**Published:** 2021-11-21

**Authors:** Sheng Zheng, Jiafeng Wang, Ning Ding, Wenwen Chen, Hongda Chen, Meng Xue, Fei Chen, Jiaojiao Ni, Zhuo Wang, Zhenghua Lin, Haiping Jiang, Xiangrui Liu, Liangjing Wang

**Affiliations:** 1 Department of Gastroenterology, The Second Affiliated Hospital of Zhejiang University School of Medicine, 88 Jiefang Road, Hangzhou, 310009 Zhejiang China; 2 Department of Pharmacology, Zhejiang University School of Medicine, Hangzhou, 310058 China; 3 Institute of Gastroenterology, Zhejiang University, Hangzhou, 310058 China; 4 Cancer Center, Zhejiang University, Hangzhou, 310058 China; 5 Department of Medical Oncology, The First Affiliated Hospital of Medical School of Zhejiang University, Hangzhou, 310016 China

**Keywords:** Gastric cancer, Cancer-associated fibroblasts, Tumor microenvironment, Polymeric prodrug, Triptolide, SN38

## Abstract

**Background:**

The prognosis of patients with advanced gastric cancer (GC) remains unsatisfactory owing to distant metastasis and resistance to concurrent systemic therapy. Cancer-associated fibroblasts (CAFs), as essential participators in the tumor microenvironment (TME), play a vital role in tumor progression. Thus, CAFs-targeting therapy is appealing for remodeling TME and sensitizing GC to conventional systemic therapy.

**Methods:**

Amphiphilic SN38 prodrug polymeric micelles (PSN38) and encapsulated the hydrophobic esterase-responsive prodrug of Triptolide (TPL), triptolide-naphthalene sulfonamide (TPL-nsa), were synthesized to form PSN38@TPL-nsa nanoparticles. Then, CAFs were isolated from fresh GC tissues and immortalized. TPL at low dose concentration was used to investigate its effect on CAFs and CAFs-induced GC cells proliferation and migration. The synergistic mechanism and antitumor efficiency of SN38 and TPL co-delivery nanoparticle were investigated both in vitro and in vivo*.*

**Results:**

Fibroblast activation protein (FAP), a marker of CAFs, was highly expressed in GC tissues and indicated poorer prognosis. TPL significantly reduced CAFs activity and inhibited CAFs-induced proliferation, migration and chemotherapy resistance of GC cells. In addition, TPL sensitized GC cells to SN38 treatment through attenuated NF-κB activation in both CAFs and GC cells. PSN38@TPL-nsa treatment reduced the expression of collagen, FAP, and α-smooth muscle actin (α-SMA) in tumors. Potent inhibition of primary tumor growth and vigorous anti-metastasis effect were observed after systemic administration of PSN38@TPL-nsa to CAFs-rich peritoneal disseminated tumor and patient-derived xenograft (PDX) model of GC.

**Conclusion:**

TPL suppressed CAFs activity and CAFs-induced cell proliferation, migration and chemotherapy resistance to SN38 of GC. CAFs-targeted TPL and SN38 co-delivery nanoparticles exhibited potent efficacy of antitumor and reshaping TME, which was a promising strategy to treat advanced GC.

**Graphical Abstract:**

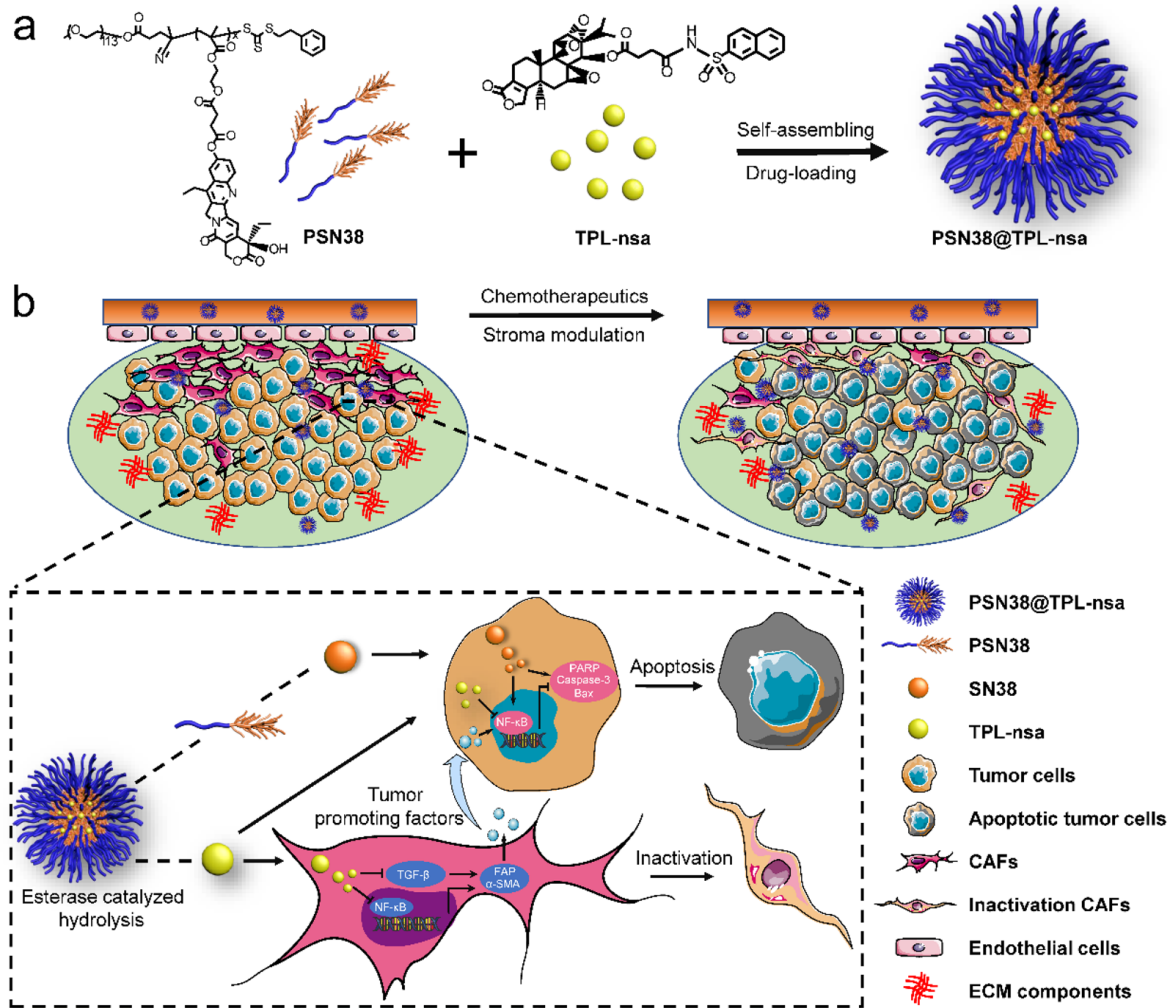

**Supplementary Information:**

The online version contains supplementary material available at 10.1186/s12951-021-01127-5.

## Background

Gastric cancer (GC) was the third most common cause of cancer-related deaths globally [1, 2]. Despite the rapid development of chemotherapy and molecular targeted therapies, the survival of advanced GC patients was dismal owing to distant metastasis and resistance to concurrent systemic therapy [3–5]. The key feature of GC is the substantial molecular heterogeneity which defined complex heterotypic interactions of cancer cells with the tumor microenvironment (TME) [6]. The TME was comprised of the extracellular matrix (ECM) where a list of non-cancer cells resides, including cancer-associated fibroblasts (CAFs), endothelial cells and diverse immune cells [7]. As a key component of the TME, CAFs not only promoted tumor growth and multidrug resistance (MDR), but also remodeling TME and mediated desmoplastic stroma [8]. CAFs dynamically involved the initiation and progression of GC, playing a role in preventing vascular access of cancer drugs through collagen production. In addition, CAFs induced chemoresistance of GC cells to cisplatin and fluoropyrimidine [9, 10], and a high CAFs proportion was associated with poorer prognosis in GC patients [11].

The existing combination regimens for advanced GC only focused on the synergistic effect on cancer cells. First-line targeted therapy with chemotherapy (trastuzumab plus platinum and fluoropyrimidine), which was the most effective combination regimen up to date, with the median overall survival of advanced GC patients increasing from 11.1 months (chemotherapy alone) to 16 months [12]. Moreover, this combination therapy was only suitable for human epidermal growth factor receptor 2 (HER2) positive GC patients, which occur in approximately 17–20% of patients with GC [13]. Targeting CAFs and reshaping the TME has been proved a promising strategy for treating advanced cancers [14]. TPL is a natural compound isolated from Chinese herb which was reported to revert the activated CAFs to the quiescent state and remodel TME to increased drug delivery into the tumor [15, 16]. The water-soluble prodrug of TPL (Minnelide) is currently in Phase II clinical trials for patients with advanced pancreatic cancer and Phase I clinical trials (combination with Paclitaxel) for the treatment of several advanced solid tumors (NCT03117920, NCT01927965, NCT04896073). However, due to its potential toxicity, rapid clearance, and low bioavailability, the clinical application of Minnelide remains an ongoing problem [17]. In addition, current combination regimens of small-molecule cytotoxic drugs and stroma-modifying agents only achieved limited improvements in clinical trials, probably due to unsatisfied pharmacokinetics and bio-distribution in tumors [18, 19]. Thus, novel synergistic strategies are urgently needed.

In recent years, the emergence of nanotechnology has provided advanced opportunities for cancer treatment [20]. Drugs can be encapsulated into nanoparticles’ inner core or loaded onto the surface of the nanocarriers and finally released after active or passive targeting to tumor sites [21]. Traditional nanocarriers were generally formed by inorganic nanoparticles such as carbon nanotube [22], grapheme [23], silica [24], and metal-based nanomaterials [25, 26], as well as organic nanoparticles including polymeric micelles nanoparticles [27], dendrimers [28], and liposomes [29]. These nanoparticles-based drug delivery systems can normalize the pharmacokinetics of loaded drugs and improve the antitumor efficacy [30]. Besides, nanoparticles can provide a platform for carrying different therapeutic agents to achieve effective combination therapy [31]. For example, CPX-351 was a liposomal nanoparticle encapsulating cytarabine and daunorubicin in an optimized 5:1 ratio, which can significantly improve the prognosis of acute myeloid leukemia (AML) [32].

**Scheme 1:**
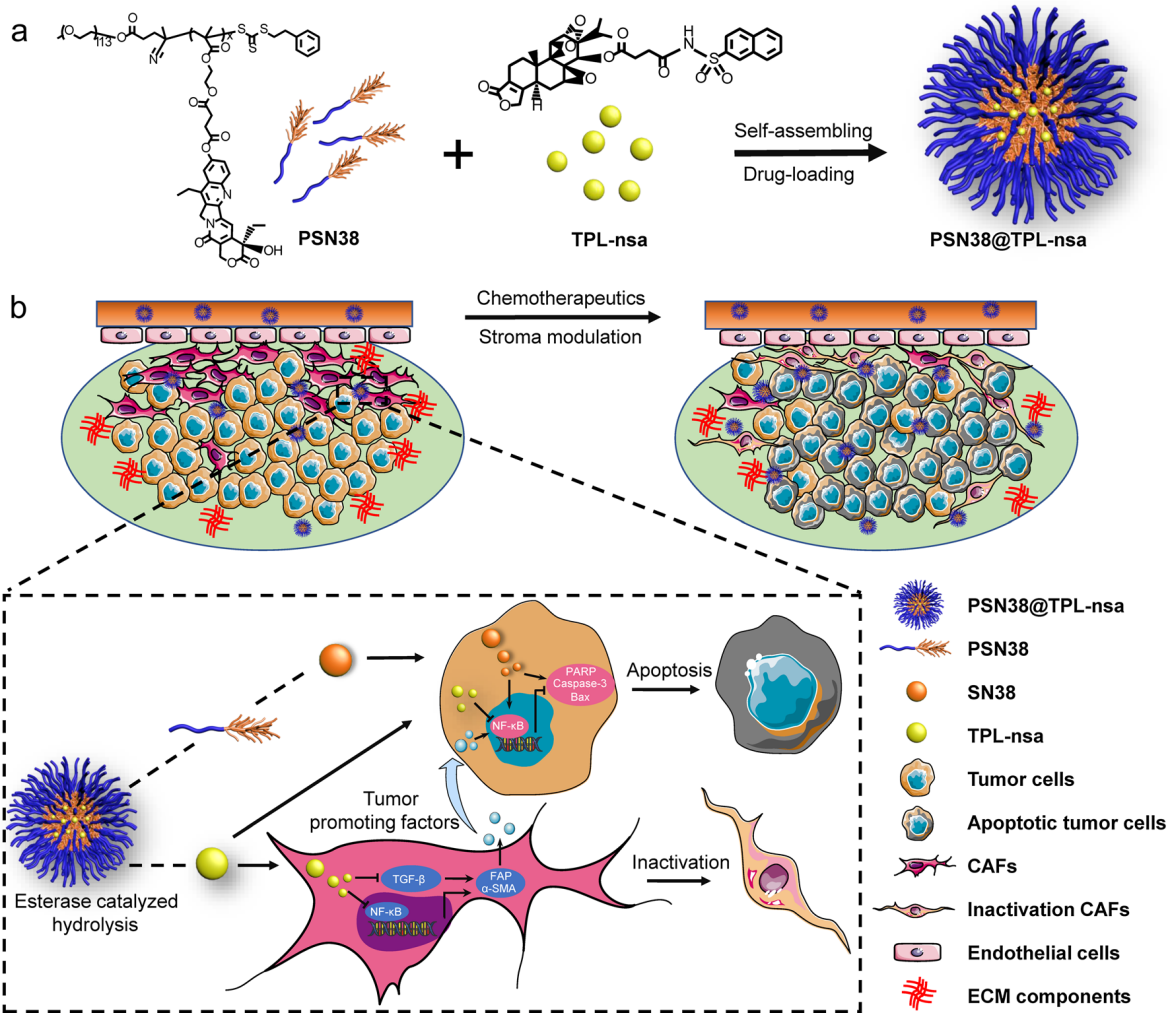
Schematic overview of the composition and synergistic mechanisms for the co-delivery nanoparticles in the fibroblast-enriched microenvironment of gastric cancer (GC). **a** A scheme illustrating the preparation of PSN38@TPL-nsa nanoparticles, including self-assembling and drug loading. **b** Schematic illustration of PSN38@TPL-nsa for synergistic therapy against GC by inactivation cancer-associated fibroblasts (CAFs) and sensitized GC cells to SN38

In this study, we first developed a polymeric nanocarrier for the co-delivery of TPL plus a potent anticancer chemotherapeutic agent SN38, which is the active form of irinotecan (CPT-11, the second-line chemotherapy for unresectable GC). The amphiphilic polymers prodrug PEG_5K_-P(MMESSN38)_5K_ (PSN38) with hydrophobic SN38 inner cores were constructed, where esterase-responsive prodrug of TPL, triptolide-naphthalene sulfonamide (TPL-nsa), was encapsulated to form PSN38@TPL-nsa nanoparticles. We found that TPL not only inactivated CAFs and reversed CAFs-induced tumor cell progression, but also sensitized GC cells to chemotherapy through attenuated NF-κB activation, suggesting its potential role in antitumor and reshaping TME via targeting CAFs in GC. PSN38@TPL-nsa administration exhibited potent antitumor efficacy and reshaped TME in CAFs-rich peritoneal disseminated tumor and patient-derived xenograft (PDX) model of GC. Mechanistically, PSN38@TPL-nsa resulted in remarkable stromal disruption effect, evidenced by significant reduction of collagen, fibroblast activation protein (FAP) and α-smooth muscle actin (α-SMA) (Scheme 1). Collectively, we proposed a nanoparticle-mediated drug co-delivery platform to integrate chemotherapy and TME-remodel as an effective treatment for advanced GC.

## Results

### Design, optimization, and characterization of PSN38@TPL-nsa nanoparticles

**Fig. 1:**
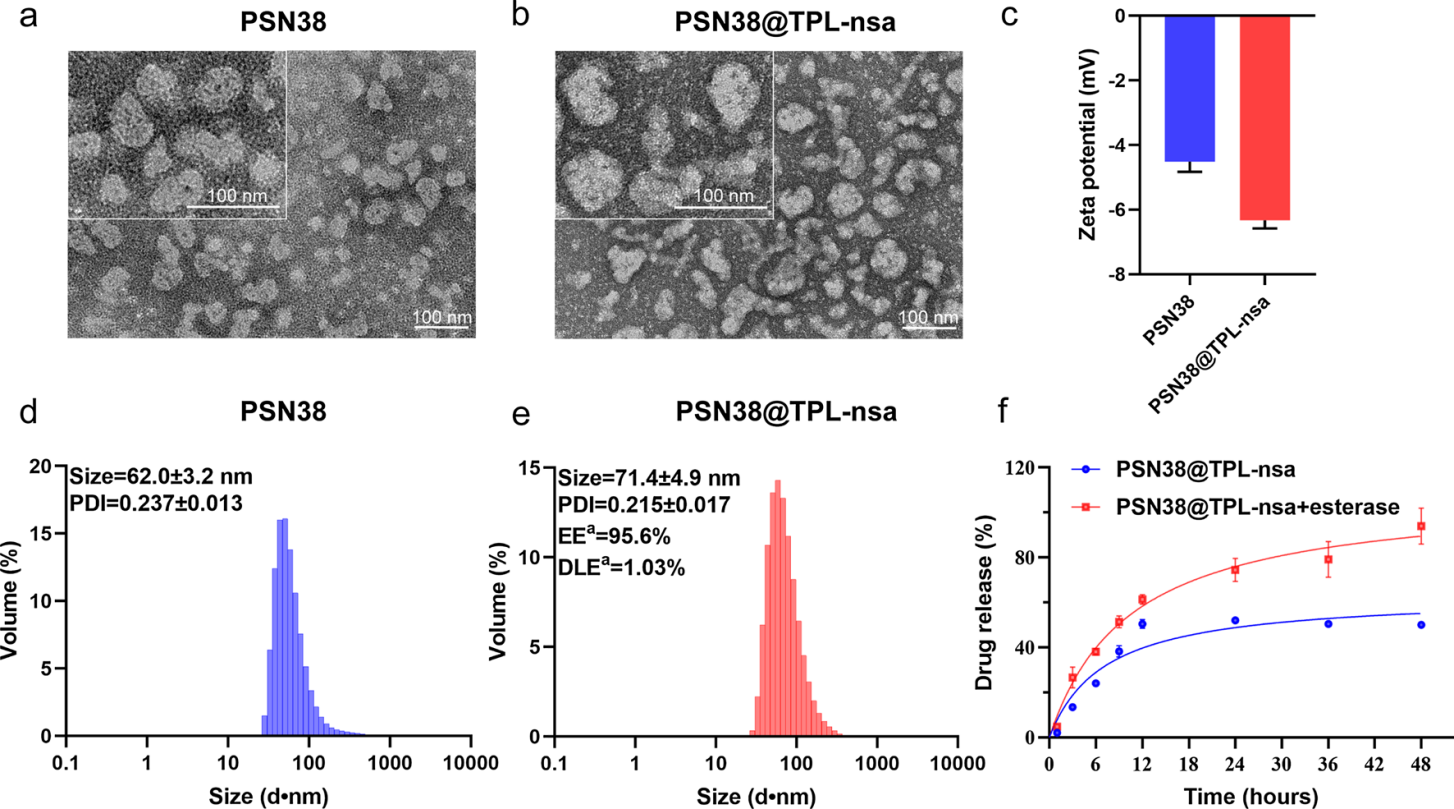
Characterization of PSN38 and PSN38@TPL-nsa nanoparticles. **a** Representative transmission electron microscopy (TEM) images of PSN38 nanoparticles. **b** Representative TEM images of PSN38@TPL-nsa nanoparticles. **c** Zeta potential of PSN38 and PSN38@TPL-nsa nanoparticles. **d** Hydrodynamic diameters and polydispersity index (PDI) of PSN38. **e** Hydrodynamic diameters, PDI and drug encapsulation efficiency of PSN38@TPL-nsa. ^a^EE (encapsulation efficiency) and DLE (drug loading efficiency) of TPL-nsa were determined via HPLC. **f** TPL-nsa release profiles in PBS with/without porcine liver esterase

Despite unique anti-cancer bioactive properties, the clinical application of TPL was drastically limited due to its toxicity and poor water solubility [33]. Here, we first synthesized amphiphilic SN38 prodrug polymer PEG_5K_-P(MMESSN38)_5K_ (PSN38) which was reported in our previous study [34, 35], to investigate whether PSN38 could encapsulate TPL into the hydrophobic inner core (Additional file 2: Fig. S1a-d). Fourier transforms infrared (FT-IR) spectra and ^1^H nuclear magnetic resonance (NMR) spectrum confirmed the successful synthesis of PSN38 (Additional file 2: Fig. S2 and Additional file 2: Fig. S3a–d). However, the incorporation of TPL into the nanoparticles did not occur (encapsulation efficacy < 10%, drug loading efficacy < 1%, data not shown). Next, we attempted several common liposome systems (DMPC, DPPC, DSPC and lecithin with different proportions of cholesterol). Similar results revealed none of them could effectively load TPL (encapsulation efficacy < 20%, drug loading efficacy < 1%, data not shown). Chao Kong et al. [36] reported the encapsulation efficacy of TPL in non-pH-sensitive micelles (NPSM) or ultra-pH-sensitive micelles (UPSM) was less than 20% due to the hydrophilicity of TPL and a hydrophobic esterase-responsive prodrug of triptolide, triptolide-naphthalene sulfonamide (TPL-nsa), designed with higher LogP could be successfully encapsulated in the hydrophobic inner core of nanoparticles with high encapsulation efficacy (> 90%). Then, we synthesized TPL-nsa (Additional file 2: Fig. S1e and Additional file 2: Fig. S3e) and PSN38 micelle loaded TPL-nsa to form PSN38@TPL-nsa nanoparticles with the encapsulation efficacy of 95.6% by the thin-film preparation method (Fig. 1e). Transmission electron microscopy (TEM) images and dynamic light scattering (DLS) analysis exhibited PSN38@TPL-nsa uniform nanostructures, with average diameters of 71.4 nm and zeta potentials of -6.33 mv (Fig. 1a–d). In vitro drug release of TPL-nsa in the existence of esterase was much faster than that without esterase as the PSN38 polymer containing phenolic ester structure also had the esterase-responsive property which could achieve tumor-specific drug release due to the high esterase concentration in tumor tissue (Fig. 1f)[37].

### CAFs was upregulated in GC tissues and associated with poorer prognosis

**Fig. 2:**
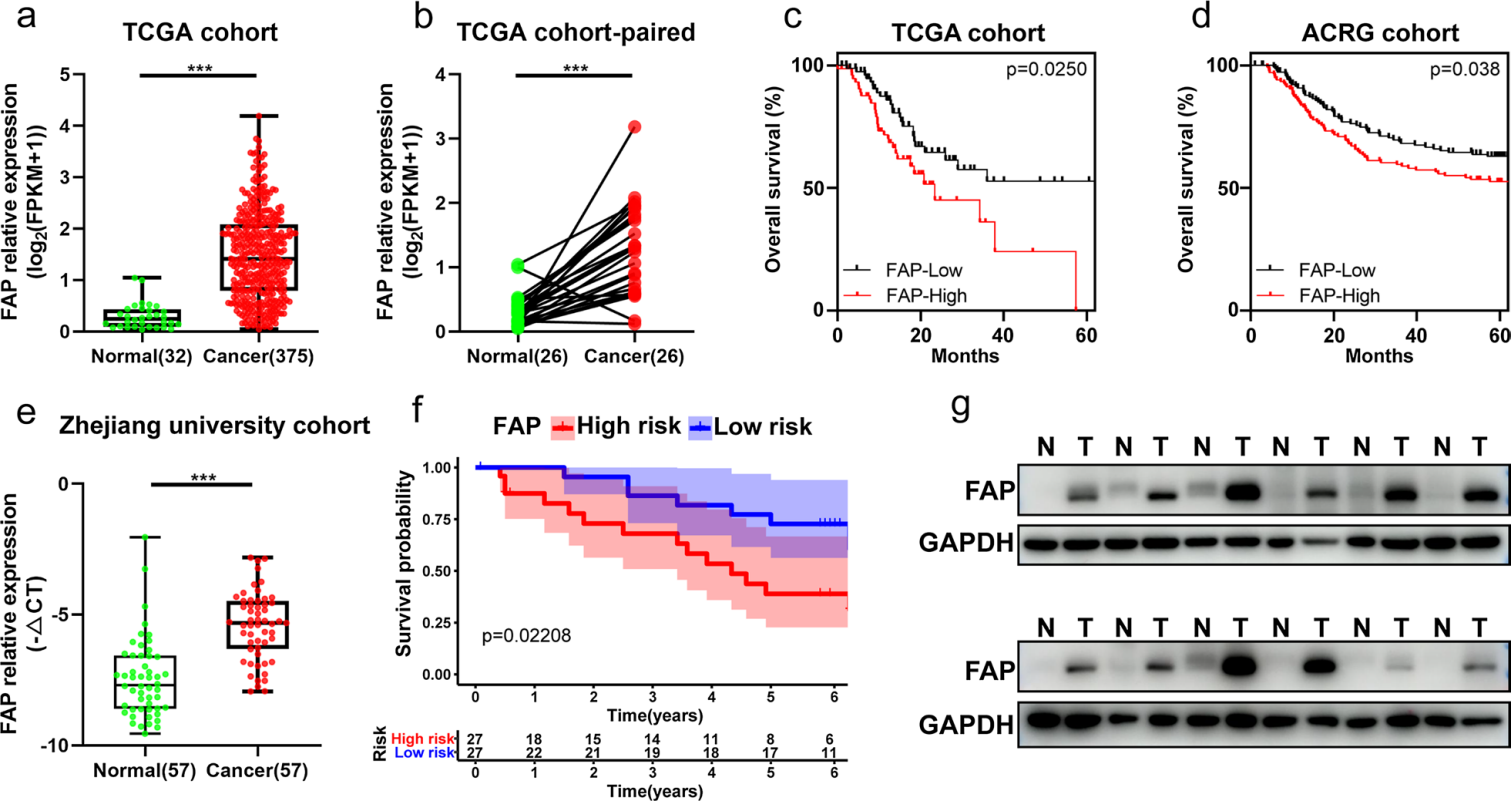
Role of CAFs in GC. **a**, **b** The RNA expression of fibroblast activation protein (FAP) between GC samples and normal gastric tissues using TCGA cohort data using unpaired (**a**) and paired (**b**) Student’s T test, respectively. **c**, **d** Kaplan–Meier overall survival analysis of GC patients in relation to FAP expression levels in TCGA cohort (**c**) and ACRG cohort (**d**). **e**, **f** The mRNA expression (**e**) and Kaplan–Meier overall survival (**f**) analysis of FAP in the GC and normal tissues using Zhejiang university cohort data. **g** Protein expression of FAP in 12 pairs of GC and adjacent normal gastric tissues by western blot analysis. (***p < 0.001)

FAP has been widely recognized as a marker of CAFs [38]. In order to further verify the role of CAFs in GC, we analyzed the expression level of FAP in The Cancer Genome Atlas (TCGA) GC cohort. The results showed that FAP was significantly upregulated in GC tissues compared with normal tissues (Fig. 2a, b). Kaplan–Meier overall survival analysis indicated that patients with the higher expression level of FAP had a poorer prognosis (Fig. 2c). Similar results were observed in both Asian Cancer Research Group (ACRG) cohort (Fig. 2d) and Zhejiang university cohort (Fig. 2e, f). In addition, we collected 12 pairs of GC and adjacent normal gastric tissues to analyze the expression of FAP. FAP proteins expression level was significantly upregulated in tumor tissues, which was consistent with previous results (Fig. 2g).

### CAFs enhanced the GC tumor formation and metastasis

Three strains of CAFs and their paired normal associated fibroblasts (NAFs) were successfully isolated and named as follows: CAF1/NAF1, CAF2/NAF2, and CAF3/NAF3. The morphologic of CAFs and NAFs showed spindle or multi-polar morphotypes (Additional file 2: Fig. S4a). To determine the identity of the primary cells, immunofluorescent staining and western blot were performed. Results showed that the FAP and α-SMA were highly expressed in the three strains of CAFs (Additional file 2: Fig. S4b). Western blot results confirmed that the expression of FAP and α-SMA in CAFs was significantly higher than that of NAFs, and which were less expressed in GC cells and gastric epithelial cells (Additional file 2: Fig. S5). In order to facilitate longtime and systematical investigation on the interaction between CAFs and GC cells, we immortalized CAFs via lentivirus-mediated stable transfection (Additional file 2: Fig. S6a). Immortalized CAFs stably expressed red fluorescent protein (RFP) after puromycin selection (Additional file 2: Fig. S6b). These immortalized CAFs were successfully passaged over 40 generations without any evidence of decay.

Conditioned medium (CM) from CAFs or NAFs were co-cultured with MKN45 and BGC-823 GC cells, and then cell proliferation and migration assays were performed (Additional file 2: Fig. S7a). The results showed that CAFs could significantly enhance the proliferation and migration ability of GC cells compared with NAFs (Additional file 2: Fig. S7b–d). To determine the effect of CAFs in vivo, xenografted tumors harboring only MKN45 cells or with MKN45 cells plus CAFs were conducted (Additional file 2: Fig. S8a). We found that the CAFs could significantly promote tumor growth and enhance the expression of FAP and α-SMA (Additional file 2: Fig. S8b–d). Similarly, in the peritoneal metastasis model of GC, the supplement with CAFs could significantly promote the metastatic ability of BGC-823 (Additional file 2: Fig. S8e–g). These results suggested that CAFs enhanced the tumorigenesis and metastasis of GC cells.

### TPL reversed the CAFs-induced GC cells proliferation, migration and chemotherapy resistance

**Fig. 3:**
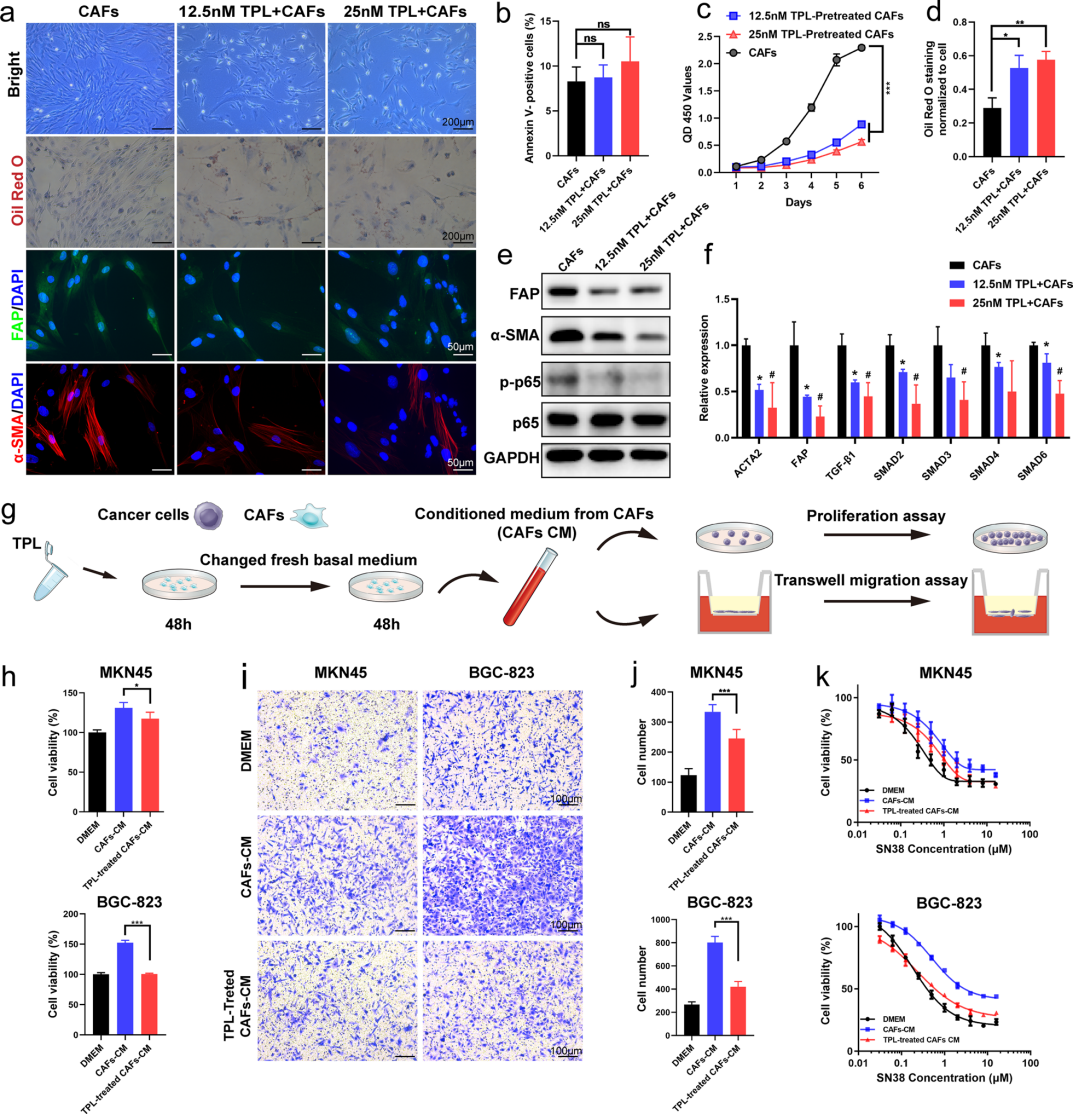
Low dose of TPL inactivated CAFs and inhibited CAFs-induced cell proliferation, migration and chemotherapy resistance of gastric cancer cells. **a** Representative image of bright photo, Oil Red staining and immunofluorescence staining of FAP and α-smooth muscle actin (α-SMA) analysis of CAFs in each group. **b** Statistical analysis of apoptotic CAF proportion treated with TPL for 48 h. **c** The proliferative abilities of CAFs after treatment with 12.5 nM or 25 nM TPL determined by CCK-8 assay. **d** Quantitative analysis of Oil red staining for CAFs treated with different concentrations of TPL for 48 h. **e** Western Blot analysis of FAP, α-SMA and NF-κB/p65 in CAFs. **f** The mRNA expression of FAP, α-SMA and genes (TGF-β1, SMAD2,3,4,6) involved in the TGF-β pathway of CAFs treated with 12.5 nM or 25 nM TPL. *, control versus 12.5 nM TPL treatment; #, control versus 25 nM TPL treatment. **g** The diagram of MKN45 and BGC-823 cells incubated with conditioned medium (CM) derived from CAFs or TPL-treated CAFs. **h** Proliferation of MKN45 and BGC-823 cells incubated with CM derived from CAFs or TPL-treated CAFs was assessed by CCK-8 assay. **i**, **j** Migration of MKN45 and BGC-823 cells incubated with CM derived from CAFs or TPL-treated CAFs. Representative images were shown (**i**) and migrated cells were counted (**j**) via Photoshop CC2019 software. **k** In vitro antitumor efficiency of SN38 against MKN45 and BGC-823 cells, with simultaneous 48 h incubations of CM derived from CAFs or TPL-treated CAFs. All data were presented as mean ± SD. Unpaired Student’s t-test was used to analyze the statistical difference. (*p < 0.05; **p < 0.01; ***p < 0.001)

To evaluate the effect of TPL on CAFs, we used a low dose of 12.5 nM and 25 nM to treat CAFs. The results showed that TPL could decrease the CAFs cells proliferation, while not affect the percentage of apoptotic cells (Fig. 3b, c). Besides, CAFs was appeared in broad and flat shapes and increased with lipid accumulation by stained Oil Red O after TPL treatment (Fig. 3a, d). Results demonstrated that TPL inhibited the expression of FAP and α-SMA by western blot, immunofluorescent staining and quantitative polymerase chain reaction (qPCR) assays (Fig. 3a, e, f). The activation of CAFs was associated with the activation of the NF-κB and TGF-β signaling pathways [15, 39]. Our results confirmed that NF-κB activation protein phospho-p65 (p-p65) was significantly decreased in CAFs treated with TPL (Fig. 3e). In addition, the mRNA expression level of TGF-β1 and its effectors pathway (SMAD2–6) was significantly decreased (Fig. 3f). Then we used CM derived from CAFs or TPL-treated CAFs to co-cultured with MKN45 and BGC-823 GC cells for proliferation, migration and SN38 cytotoxicity assays (Fig. 3g). The results showed that CM from TPL-treated CAFs reversed the effect of CAFs-induced GC cells proliferation and migration (Fig. 3h–j). Cell cytotoxicity assays showed that TPL-treated CAFs could reverse CAFs-induced SN38 chemotherapy resistance in MKN45 and BGC-823 cells (Fig. 3k).

### TPL sensitized GC cells to SN38 treatment

**Fig. 4:**
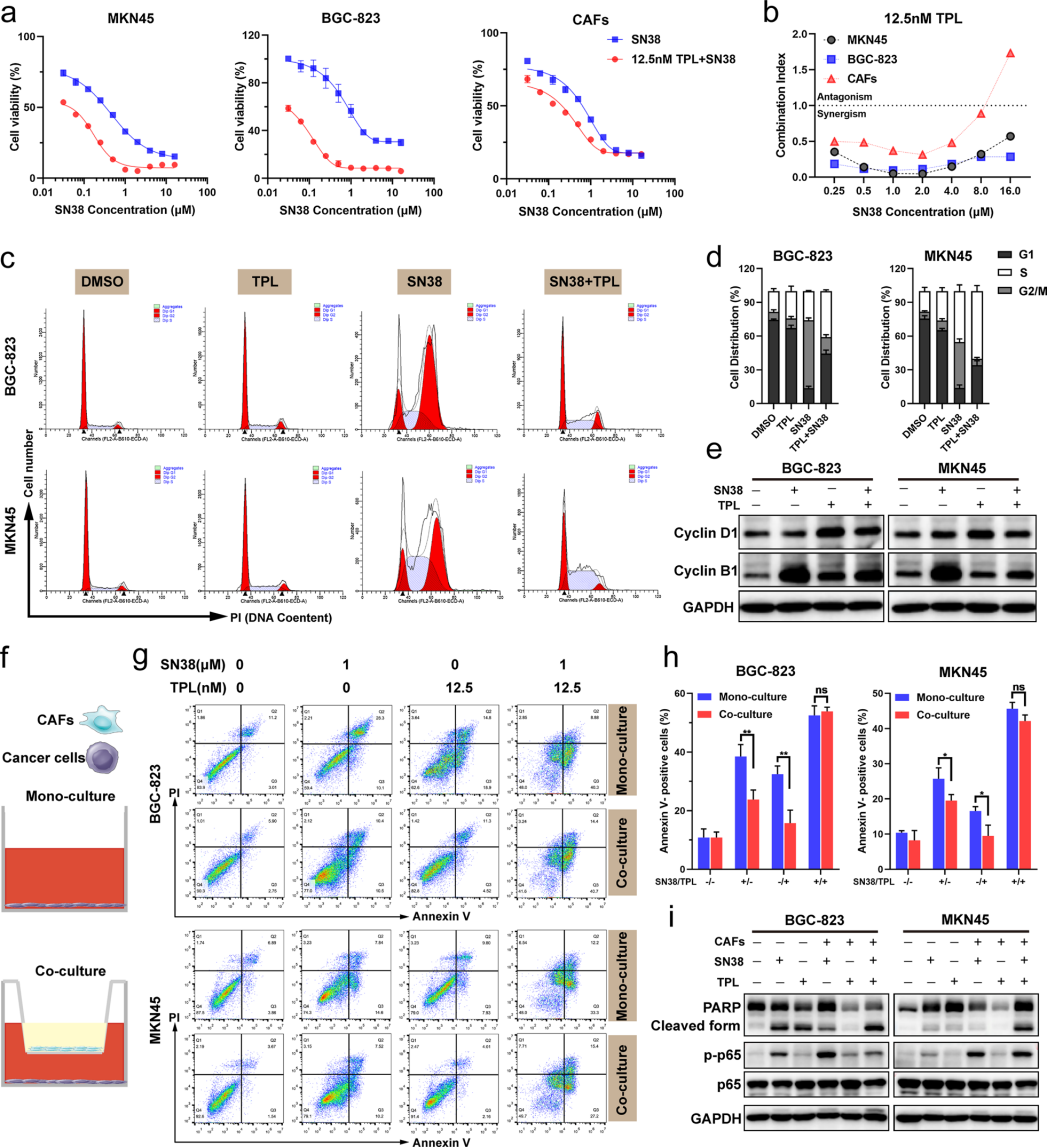
TPL sensitized GC cells to SN38 and reversed the SN38 resistance induced by co-culture with CAFs. **a** Cell viability of MKN45, BGC-823 and CAFs after treated with different concentrations of SN38 combined with/without 12.5 nM TPL for 48 h. **b** Combination index (CI) analysis of TPL and SN38 combinational therapy in MKN45, BGC-823 and CAFs. **c**, **d** Cell cycle distributions of MKN45 and BGC-823 cells after treated with TPL, SN38 or a combination of TPL and SN38 for 24 h. **e** Western Blot assay of cyclin D1 and cyclin B1 of MKN45 and BGC-823 with different treatments. **f** Illustration of mono-culture and co-culture models. In the mono-culture model, GC cells were cultured individually. Transwell chambers were utilized in the co-culture model: CAFs were cultured in the upper chamber; whereas, GC cells were cultured in the lower chamber. **g, h** Apoptotic analysis of BGC-823 and MKN45 cells after the treatment of TPL, SN38 or a combination of SN38 and TPL for 24 h in the co-culture and mono-culture model, respectively. **i** Western Blot analysis of PARP, cleaved PARP and NF-κB/p65 in the BGC-823 and MKN45 cells which were treated with TPL, SN38 or a combination of TPL and SN38 for 24 h in the co-culture model. All data are presented as mean ± SD. Unpaired student’s t-test was used to analyze the data. (*p < 0.05; **p < 0.01; ***p < 0.001)

To determine the effect of TPL on cell viability, MKN45, BGC-823 and CAFs cells were treated with indicated doses of TPL for 48 h. A dose-dependent decrease in cell viability was observed (Additional file 2: Fig. S9a). We found that the combined treatment with TPL and SN38 substantially suppressed GC cells and CAFs growth compared with SN38 alone (Fig. 4a). In order to assess this synergistic effect, the combination index (CI) value was calculated by CalcuSyn software which synergism, additivity and antagonism were defined by CI < 1, CI = 1 and CI > 1, respectively. The CI value showed that the synergistic effect in GC cells (0.04–0.6) was stronger than that in CAFs (0.3–1.7) when exposed to the combination of 12.5 nM TPL and variable concentrations of SN38 (Fig. 4b). Mechanically, the combined treatment with TPL and SN38 significantly enhanced the expression of cleaved caspase-3 and PARP in GC cells, while not in CAFs (Additional file 2: Fig. S9b). These results suggested that TPL at a subtoxic concentration enhanced the suppressive effect with SN38-treatment on GC cells proliferation and activating a caspase-involved apoptotic pathway.

To explore the synergistic mechanism of combination treatment with TPL and SN38, we used Flow cytometry to determine the cell cycle distribution in GC cells. Results indicated that the treatment of SN38 obviously induced cell cycle arrest at the G2/M phase. Combined treatment could reduce SN38-induced G2/M arrest and increase the distribution of GC cells in the S phase (Fig. 4c, d). SN38 is a chemotherapy agent that causes S phase specific cell killing by poisoning topoisomerase I (Topo I) in the cells [40]. SN38 treatment alone could increase the expression of cyclin B1 protein, while this effect was reversed when combined with TPL (Fig. 4e).

We then constructed cell co-culture models to mimic the CAFs-enriched microenvironment in vitro and measured SN38-induced apoptosis in BGC-823 and MKN45 cells (Fig. 4f). Co-culture with CAFs reduced the percentage of apoptotic BGC-823 cells from 32.43 ± 2.822% to 23.8 ± 3.242% (*p* = 0.0254) by SN38 treatment. TPL combined with SN38 could reverse the chemotherapy resistance of BGC-823 cells induced by CAFs, and increase the proportion of apoptotic cells (52.5 ± 3.239%) (Fig. 4g, h). The expression of NF-κB activation protein p-p65 increased, while cleavage of PARP decreased when treated with SN38 as compared to the mono-cultured system. Combined treatment in the co-cultured system had an effect on down-regulation with p-p65 and increase cleaved PARP, indicating that TPL effectively inactivated NF-κB signaling and reversed CAFs induced SN38 resistance (Fig. 4i).

### PSN38@TPL-nsa suppressed the metastatic intraperitoneal tumors containing CAFs

**Fig. 5:**
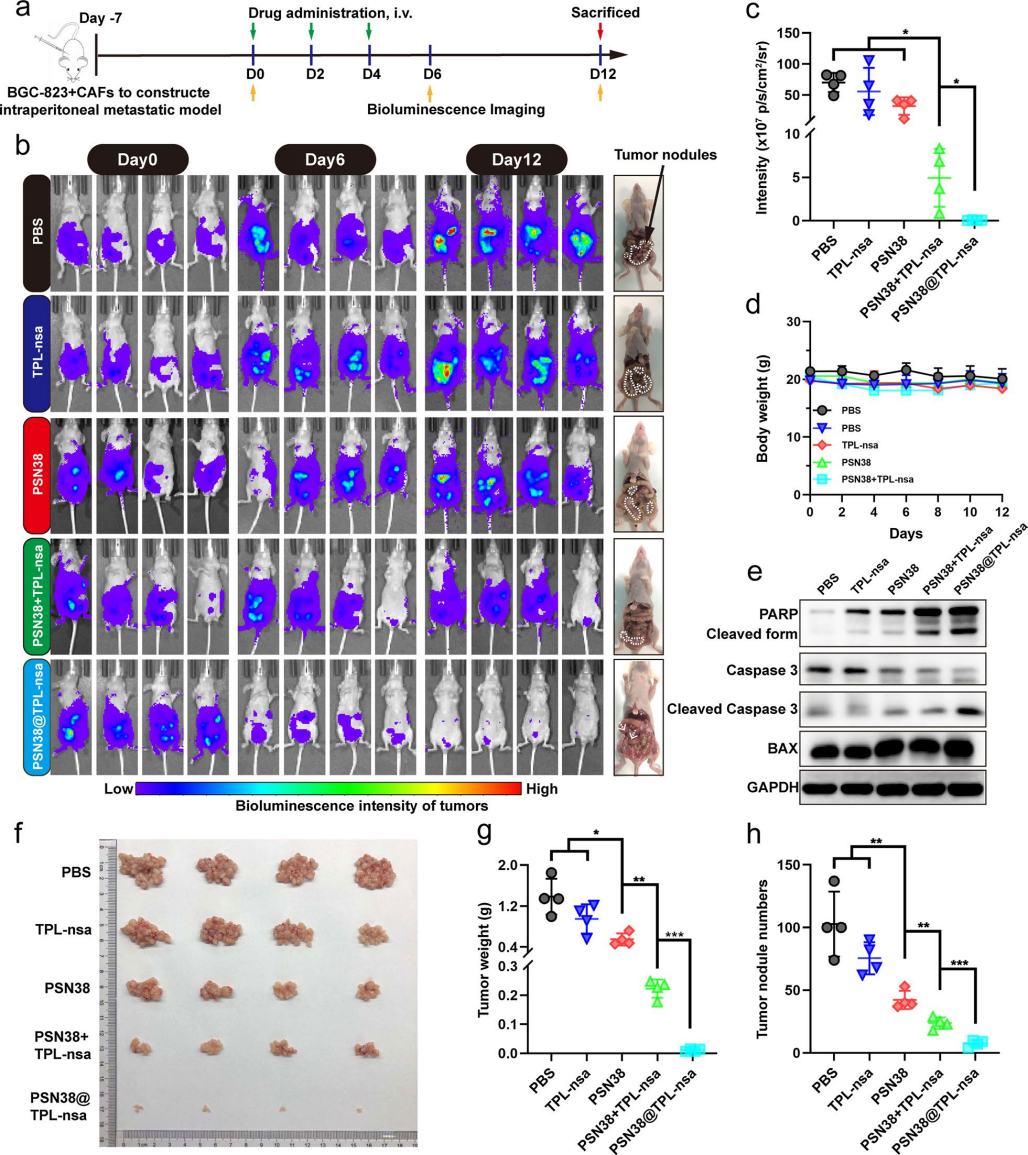
PSN38@TPL-nsa antitumor efficiency in the CAFs-containing intraperitoneal tumor model. **a** Establishment of CAFs-containing intraperitoneal tumor model and schedule of in vivo antitumor experiments. **b** In vivo bioluminescence imaging of the mice after intravenous injections of PBS, TPL-nsa, PSN38, PSN38 + TPL-nsa and PSN38@TPL-nsa (n = 4). White dotted lines and arrows indicated the tumor nodules. **c** Quantitative analysis of bioluminescence imaging intensity on Day 12. **d** Body weight variation in the tumor-bearing mice during the experimental period (n = 4). **e** Western blotting analysis of PARP, caspase-3 and BAX family proteins of tumors in the different groups at the end of experiments. **f** Dissected intraperitoneal tumor nodules after humanitarian execution (n = 4). **g**, **h** The intraperitoneal tumor weights (**g**) and nodule numbers (**h**) in each group were statistically analyzed. All data are presented as mean ± SD. Unpaired Student’s t-test was used to analyze the data. (*p < 0.05; **p < 0.01; ***p < 0.001)

To evaluate the tumor suppressing effect of the PSN38@TPL-nsa, we developed the intraperitoneal tumor model containing CAFs by co-injection of luciferase stably expressing BGC-823 (BGC-823-luci) cells and CAFs in the abdominal cavity of nude mice (Fig. 5a). The dynamic capture of in vivo imaging system (IVIS) images by in vivo luciferase bioluminescence imaging (BLI) was measured. Results showed that TPL-nsa or PSN38 treatment had weak effects on tumor metastasis (Fig. 5b, c). PSN38@TPL-nsa group significantly eliminated abdominal metastatic tumors and elicited an excellent suppression of tumor growth than PSN38 + TPL-nsa free drug mixture (Fig. 5b–d). PSN38@TPL-nsa treatment enhanced the expression of cleaved caspase-3 and PARP (Fig. 5e), indicating the synergistic effect of TPL-nsa loaded SN38 nanoparticles on cell apoptosis. In addition, PSN38@TPL-nsa group exhibited less scattered tumor nodules on the mesentery compared with other groups (Fig. 5f) and better antitumor efficacy both in tumor weight and tumor nodule numbers (Fig. 5g, h).

### PSN38@TPL-nsa exhibited antitumor efficacy on GC patient-derived xenografts

**Fig. 6:**
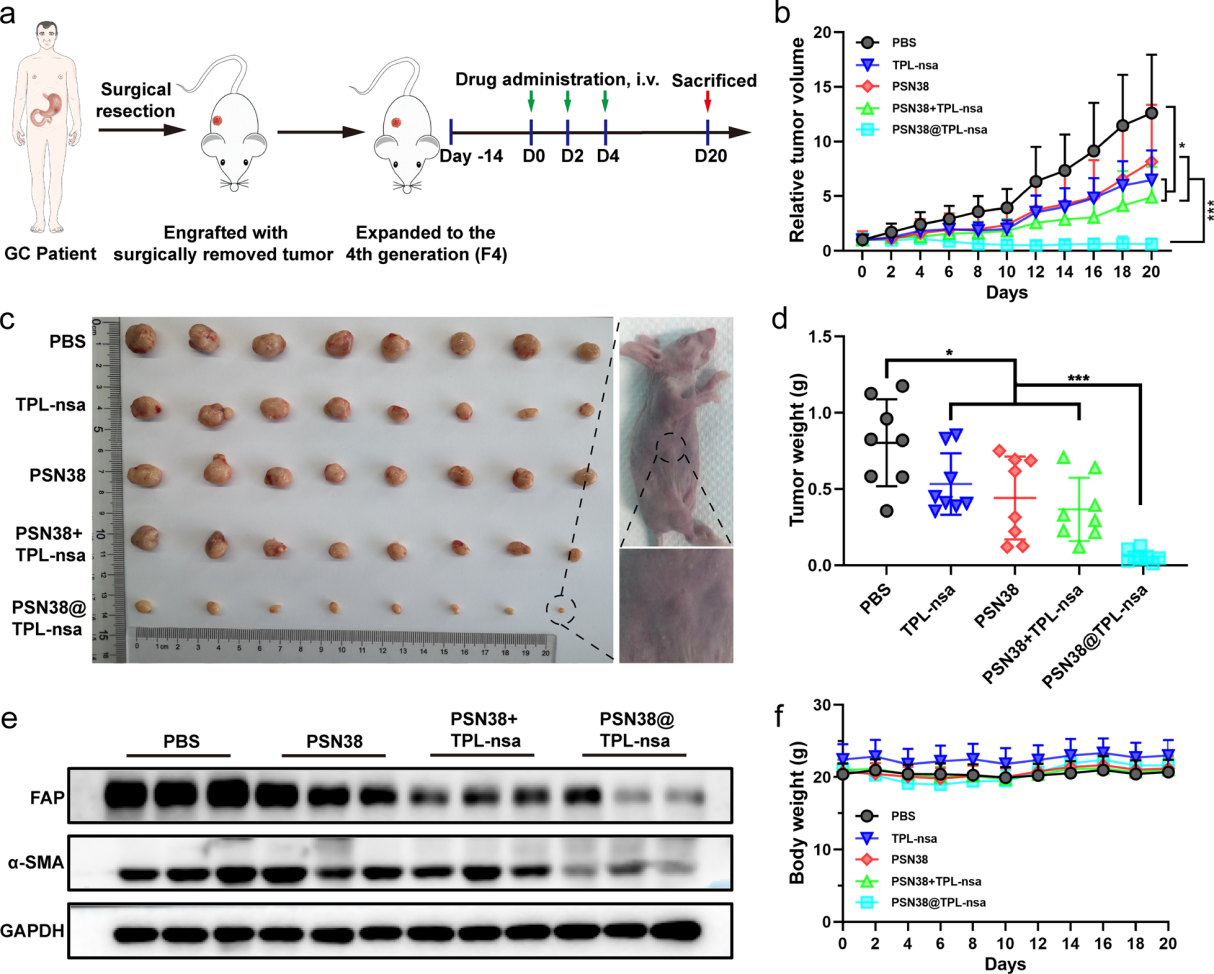
The antitumor efficiency of PSN38@TPL-nsa in GC PDX model. **a** Therapeutic schedule of treatments of mice with GC PDX model. **b** The tumor growth curves after intravenous injection of PBS, TPL-nsa, PSN38, PSN38 + TPL-nsa and PSN38@TPL-nsa (n = 8). **c** Images of the excised tumor in the different groups at the end of in vivo antitumor evaluation (n = 8 for each group). **d** The average tumor weight of excised tumors in each group at the experimental endpoint. **e** Western blot assay of FAP and α-SMA of tumor tissues at the end of in vivo antitumor evaluation. **f** Body weight variation in the tumor-bearing mice during the experimental period (n = 8). All data are presented as mean ± SD. Student’s t-test was used to analyze the data. (*p < 0.05; **p < 0.01; ***p < 0.001)

GC is a disease with substantial molecular heterogeneity, and the response to anti-cancer therapy varies. The TME and the relative proportion of cancer cells and stromal cells are both maintained in the PDX models [41]. We constructed PDX models to explore whether our strategy of co-delivery PSN38@TPL-nsa was promising for GC patients. PBS, TPL-nsa, PSN38, PSN38 + TPL-nsa and PSN38@TPL-nsa were intravenously injected every other day for 3 times (Fig. 6a). TPL-nsa, PSN38 or PSN38 + TPL-nsa showed limited antitumor efficacy with a fast tumor rebound after treatment-free period (Fig. 6b, c). PSN38@TPL-nsa exhibited excellent antitumor efficiency resulting in a tumor-growth inhibition rate (TIR %) of 92.84 ± 4.8% compared that of PSN38 + TPL-nsa (54.36 ± 25.82%) at the end of experiment (Fig. 6d). PSN38@TPL-nsa treatment suppressed the expression of FAP and α-SMA in tumor tissues (Fig. 6e).

For biosafety evaluation, organs were excised from PDX model mice on day 20 and pathologically analyzed. There was no notable damage in all treatment groups (Additional file 2: Fig. S10c). Administration with PSN38, TPL-nsa, PSN38 + TPL-nsa or PSN38@TPL-nsa had no effect on the body weight of mice (Fig. 6f). Blood routine and blood biochemical examinations were within a normal range in PSN38@TPL group, which indicated PSN38@TPL-nsa at current therapeutic dosages didn’t cause severe or permanent myelosuppression and liver and kidney damages (Additional file 2: Fig. S10a, b). These data supported the safety profile of PSN38@TPL-nsa.

### PSN38@TPL-nsa reshaped tumor microenvironment in vivo

**Fig. 7:**
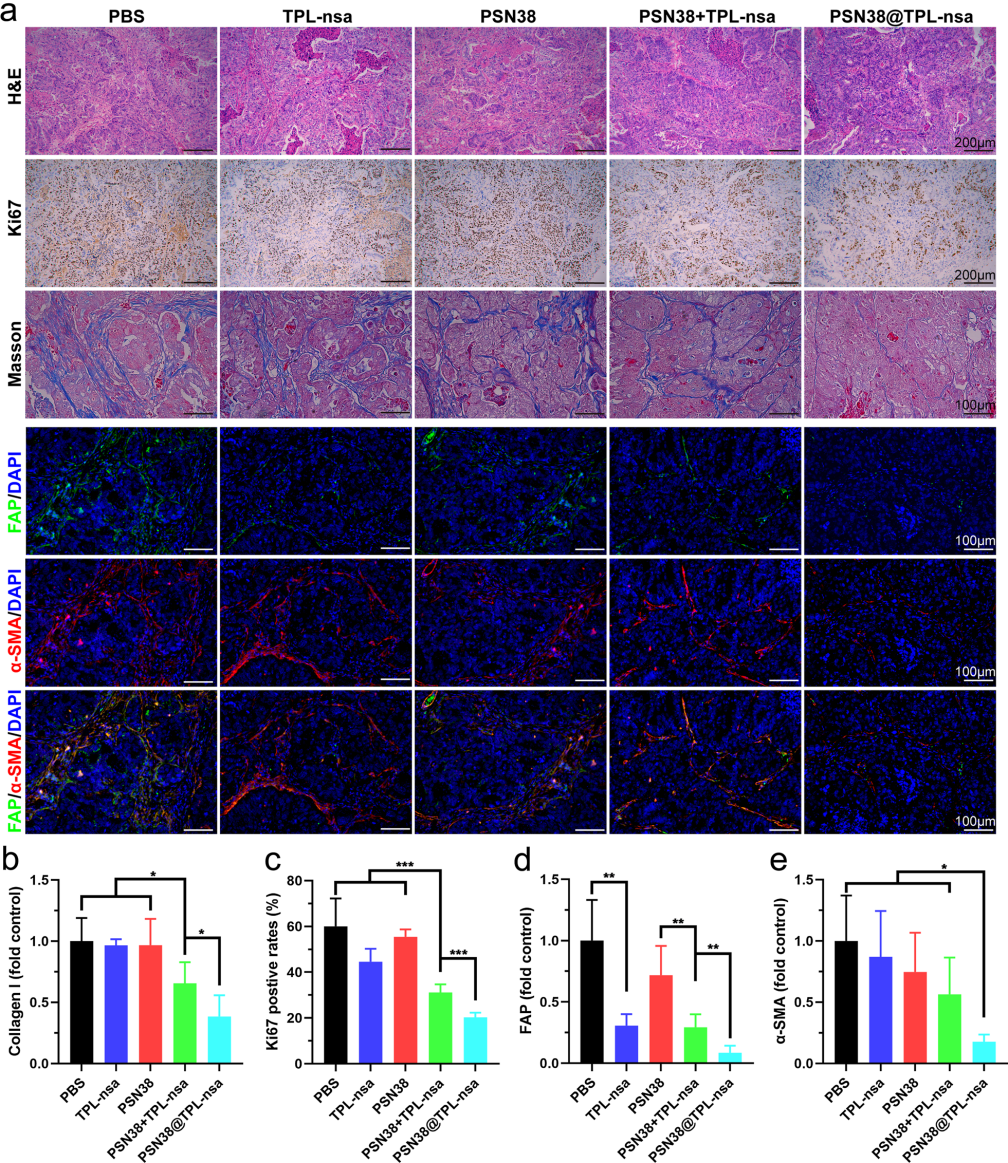
PSN38@TPL-nsa remodeled GC microenvironment in vivo. **a** Representative image of H&E staining, IHC staining (Ki67), Masson's trichrome and Immunofluorescence staining (FAP and α-SMA) of tumors at the experimental endpoint. **b**–**e** Quantitative analysis of collagen content, Ki67, FAP and α-SMA using Image J from 3 to 5 randomly selected microscopic fields, respectively. All data are presented as mean ± SD. Student’s t-test was used to analyze the data. (*p < 0.05; **p < 0.01; ***p < 0.001)

The dense ECM components in TME are mainly secreted and regulated by CAFs, among which collagen are the key components serving as the physical barrier for drug delivery [42]. We next investigated the effect of PSN38@TPL-nsa on modulation of the TME in PDX model. Disordered glandular structure with numerous apoptotic cells and decreased stromal components were observed in the PSN38@TPL-nsa treated group, compared with the regular glandular structure tightly-packed stromal cells in the other groups (Fig. 7a). Immunohistochemistry (IHC) staining revealed that PSN38@TPL-nsa efficiently suppressed the expression of Ki67 and collagen I, which were markers for tumor proliferation and main components of tumor stroma respectively (Fig. 7b, c). Furthermore, the expression of FAP and α-SMA was remarkably downregulated in PSN38@TPL-nsa group by immunofluorescence staining (Fig. 7d, e). Taken together, nanoparticle-based synergistic therapy of PSN38@TPL-nsa exhibited high efficacy of modulation with the TME.

## Discussion

Currently, the standard choice of sequential lines of chemotherapy for patients with advanced GC shows limited effectiveness and the median overall survival is merely 11 months [43, 44]. Various molecularly targeted agents have been proposed to enhance the survival rate of advanced GC, but most clinical trials did not show a survival benefit [45, 46]. Accumulating evidence suggested that GC progression, metastasis and chemoresistance were related to the TME, while not depending solely on cancer cell-autonomous defects. In this study, polymeric SN38 prodrug nanoparticle loading TPL-nsa resulting with reduced CAFs activity, reshaping the TME and reversing the CAFs-induced chemoresistance was efficient for anti-GC therapy.

Nanomedicines have attracted tremendous attention and extensively applied in the delivery of chemotherapeutic agents because of their advantages in improving the solubility of hydrophobic drugs, enhancing cellular uptake and alleviating off-target toxicities [47, 48]. By co‑delivering multiple active drug components, nanoparticles could facilitate synergistic anticancer effect and attenuate drug resistance, as evidenced by a number of in vivo studies (Additional file 2: Table S1). These studies provided insights into heterogeneities within tumors, which were important for potential personalized treatment [49]. In our study, we synthesized PSN38 polymeric micelles and encapsulated TPL-nsa in the hydrophobic core by self-assembly to form PSN38@TPL-nsa nanoparticles. PSN38 polymeric micelles can protect the loaded TPL-nsa from premature degradation, which helps minimize the loss of antitumor efficacy. In addition, the uniform particle size of PSN38@TPL-nsa (71.4 ± 4.9 nm) can help achieve passive aggregation at tumor sites through enhanced permeability and retention (EPR) effect. PSN38@TPL-nsa could also achieve tumor-specific drug release due to abnormal high esterase concentration in tumor sites. TPL and SN38 released from tumor site exerted excellent anti-tumor and TME remodeling effects with limited biotoxicity.

The TME, composing of non-cancerous cells and components presented in the tumor, plays decisive roles in tumor initiation, progression, metastasis and response to therapies. Among the stromal cells present in the TME, CAFs are most abundant, play an important role in TME by secreting cytokines and remodeling the ECM [14]. Therefore, as a promising therapeutic target for cancer, CAFs have attracted amounts of attentions. To better mimic the human GC microenvironment with an enrichment of CAFs, we immortalized CAFs and constructed a cellular co-culture model in vitro and GC tumor model embracing both human tumor cells and CAFs in vivo. In these models, we observed that CAFs could promote GC cells progression, SN38 resistance, and tumorigenesis. Meanwhile, some studies indicated that direct depletion of CAFs may lead to accelerated cancer progression and immunosuppression [50]. Thus, alternative strategies to deplete CAFs have been investigated, such as reducing activity of CAFs, or inducing transition to the quiescent state. In the present study, we found that TPL at low concentrations significantly inhibited the activity of CAFs (Fig. 3c) and sensitized GC to SN38 (Fig. 4a), but did not increase the apoptosis of CAFs (Fig. 3b). Besides, PSN38@TPL-nsa exhibited remarkable stromal disruption effect, evidenced by significant reduction of collagen, FAP and α-SMA. Therefore, TPL is a suitable stromal reprogramming inducer by CAFs activity reduction without increasing apoptosis.

TPL was first isolated from a perennial vinelike Chinese medicinal herb called *Tripterygium wilfordii* Hook f (TWHf) in 1972 and has attracted considerable interest due to its various biological functions, such as antitumor effect, immunosuppression and anti-inflammatory properties [51]. In previous studies, TPL exerted biological effects through targeting various pathways and genes, such as NF-κB and TGF-β pathway, MYC and IL-1 genes. NF-κB is an important signaling pathway in cell proliferation, tumor survival and drug resistance, et al. [39, 52]. Aberrant activation of NF-κB signaling pathway has been reported in a variety of tumors, playing an important role in the communication between tumor cells and CAFs. For instance, Su et al. showed that CAFs could sustain tumor cell stemness and promote tumor chemoresistance by maintaining NF-κB signaling pathway activation [53]. Moreover, sequential chemotherapy, which are commonly used in advanced GC patients, might also induce DNA damage in stromal cells, resulting in the activation of NF-κB, eventually contributing to therapeutic resistance [54]. In this study, when GC cells were co-cultured with CAFs and then treated with SN38, the expression of NF-κB activation protein p-p65 dramatically increased, which resulted in the chemoresistance to SN38 (Fig. 4h, i). TPL could inactivate CAFs and sensitized GC cells to SN38 through hampering NF-κB signal pathway in both CAFs (Fig. 3e) and GC cells (Fig. 4i), suggesting its potential antitumor efficacy in advanced GC combination therapy.

PDX models have been widely adopted as a powerful tool in the evaluation of drug efficiency owing to the fact that PDX models can retain the unique features (such as gene patterns, TME properties and responses to drug treatment) of tumors in patients [55]. In this study, a PDX model was established to verify the therapeutic effect of PSN38@TPL-nsa. The results showed that PSN38@TPL-nsa significantly inhibited the activity of CAFs and reduced the collagen I. Compared with SN38, TPL-nsa or the combination, PSN38@TPL-nsa was more potent to inhibit the tumor growth and remodel the TME in GC PDX model (Fig. 6b). The antitumor evaluation assay of PSN38@TPL-nsa suggested that GC patients might benefit from the SN38 and TPL combination therapy. Co-delivery SN38 and TPL to remodel TME exhibited synergistic effects and holds potential to improve therapeutics against advanced GC.

## Conclusion

TPL at a subtoxic concentration not only inactivated CAFs and reversed CAFs-induced SN38 resistance, but also sensitized GC cells to SN38 by suppressing NF-κB pathway in GC cells. We developed an esterase responsive SN38 and TPL co-delivery system (PSN38@TPL-nsa) for synergistic GC therapeutics. PSN38@TPL-nsa exhibited potent antitumor and TME remodeling efficacy in GC intraperitoneal and PDX tumor models. The PSN38@TPL-nsa nanoparticles co-delivery system provided a promising strategy for advanced GC treatment.

## Materials and methods

### Materials

SN38 was purchased from Xi'an Xindifu Science and Technology Co. (Xi'an, China). TPL was purchased from WeiFang Huazhi Science and Technology Co. (WeiFang, China). Sodium hydride and other chemical reagents for PSN38@TPL-nsa synthesis were purchased from Sigma-Aldrich, China.

### Preparation and characterization of PSN38@TPL-nsa nanoparticles

Details and methods of the synthesis of PSN38 and TPL-nsa were provided in Additional file 1. PSN38@TPL-nsa nanoparticles was prepared by thin-film evaporation and sonication method. 50 mg PSN38 and 0.5 mg TPL-nsa were dissolved in 5 ml Dichloromethane (DCM) and evaporated to form a thin film which was hydrated by 5 ml water. Then the solution was probe sonicated at 200 W for 15 min and ultrafiltrated (MW cutoff 10 k Da, Millipore) to remove free unencapsulated drug. To characterize the chemical compositions of synthesized copolymers, ^1^H NMR spectra were obtained using a Bruker Avance DRX-400 spectrometer (Bruker BioSpin Corporation, Billerica, MA) and FT-IR spectra were recorded using attenuated total reflectance (Nicolet iS50, Thermo Fisher, USA). Nanoparticle size and zeta potential were measured by DLS (Malvern Instruments, U.K.). The morphology of PSN38 and PSN38@TPL-nsa was observed and imaged by TEM (TECNAL 10, Philips).

### In vitro drug release of PSN38@TPL-nsa nanoparticles

PSN38@TPL-nsa nanoparticles solution was concentrated at a TPL-nsa concentration of 1.33 mg/ml. 3 ml concentrated solution with/without 60 U/ml porcine liver esterase was loaded into a dialysis bag (MWCO, 3.5 kDa) which was immersed into 30 ml PBS. Then the whole release system was shaken at 37 ℃ at 100 rpm. 0.1 ml solution was collected for high performance liquid chromatography (HPLC, Hitachi HPLC Primaide, Japan) analysis at predetermined time intervals (1, 3, 6, 9, 12, 24, 36, 48 h). For the release system with esterase, both TPL and TPL-nsa concentration was calculated to obtain the release profile as TPL-nsa could also be degraded by esterase.

### Cell culture

Gastric cancer cell lines MKN45, BGC-823, SGC7901 and AGS were obtained from the Chinese Academy of Medical Sciences and cultured in RPMI 1640 medium supplemented with 10% fetal bovine serum (FBS) and 1% antibiotics. Normal gastric epithelial cell line GES-1 was obtained from the Chinese Academy of Medical Sciences and cultured in DMEM medium with 10% FBS and 1% antibiotics. All cells were grown routinely in a monolayer culture at 37 °C in a 5% CO_2_ humidified atmosphere.

### Clinical tissue sampling

We analyzed the expression and prognosis of FAP in TCGA stomach adenocarcinoma cohort (https://portal.gdc.cancer.gov/) and ACRG cohort (GSE62254, https://www.ncbi.nlm.nih.gov/geo/). Tumor and adjacent nontumorous tissues of 57 GC patients were obtained from the Second Affiliated Hospital of Zhejiang University School of Medicine. qPCR was performed to calculate the relative expression of FAP. The clinical GC cohorts were separately dichotomized into high-risk and low‐risk subgroups. The survival curve was drawn by Kaplan–Meier method. The clinical tissue sampling study was approved by the ethics committee of The Second Affiliated Hospital of Zhejiang University School of Medicine and we got informed consent from all patients.

### Isolation CAFs and NAFs

Primary CAFs were isolated by the outgrowth method [56]. Primary GC tumor and adjacent normal samples were sterilely obtained after the surgery at The Second Affiliated Hospital, Zhejiang University School of Medicine. CAFs were isolated from their GC tissues and NAFs were isolated from their paired normal tissues, as described in Additional file 1.

### Immortalization of CAFs

The immortalization of CAFs was performed by stable transfection using viruses carrying plasmids encoding SV40 large T antigen. These plasmids also carried RFP and puromycin-resistance gene, which enabled selection with puromycin. Cells were incubated with puromycin (ABM Co., 4 μg/mL) for 48 h, and resistant clones were expanded until the cells reached terminal crisis. After crisis, CAFs clones were cultured in DMEM medium plus 10% FBS and 1% antibiotics.

### Production of conditioned medium

CM derived from CAFs or NAFs was produced using FBS-free DMEM medium according to previous literature [15]. In normal conditions, 80% confluent cells were cultured in FBS-free DMEM medium for 48 h (Additional file 2: Fig. S6a). In experiments designed to analyze the effects of TPL, 80% confluent cells were cultured in DMEM medium with 10% FBS containing 25 nM TPL for 48 h and then changed to no drug, FBS-free, DMEM medium for 48 h (Fig. 3g). The resulting CM were centrifuged for 15 min at 2500 g after collection and stored at − 80℃. Before use, the CM was thawed in 4℃ and complemented with 10% FBS.

### Cell viability assay

The cell viability was investigated using the Cell Counting Kit-8 (CCK-8), which was obtained from Meilunbio (Dalian, China). Briefly, GC cells were seeded at the density of 5000 per well in 96-well plates and incubated overnight. Then, the medium of cells was replaced with CM derived from CAFs or NAFs and incubated for 48 h. After exposure, the medium in each well was replaced with 110 μL fresh medium containing 10% CCK-8 solution and incubated for 2 h at 37 °C in the dark. The absorbance of each well was measured at 450 nm on an automatic microplate reader (Spark Cyto Brochure, USA).

For exploration the paracrine effect of CAFs on the responsiveness of GC cell lines to chemotherapy, CM derived from CAFs or TPL-treated CAFs with various concentrations of SN38 were added to 96-well plates and incubated for 48 h to examined the cell viability. For exploration of combination index (CI) of SN38 and TPL, various concentrations of TPL, SN38 or SN38 plus 12.5 nM TPL were added into 96-well plates seeded with GC cells or CAFs and incubated for 48 h. When the incubation was finished, the cell viability was measured.

### Transwell migration assay

The CM, derived from CAFs or TPL-treated CAFs, was respectively added to a 24-well plate and DMEM containing 10% FBS was set as the negative control. In each group, three repeated wells were set. 100 μL FBS-free DMEM, containing 1.0 × 10^5^ MKN45 or BGC-823 cells, was added to upper chamber and a conventional culture was conducted for 24 h. After the cells were fixed with methanol and stained with crystal violet. Under the microscope, upper, lower, central, left and right 5 view fields were selected to count the number of transmembrane cells.

### Cell cycle analysis

For the detection of changes in the cell cycle after different drug exposure, MKN45 and BGC-823 cells were seeded in 6-well plates with 2.0 × 10^5^ cells per well. After treatment with 12.5 nM TPL, 0.01 μM SN38 or 0.01 μM SN38 plus 12.5 nM TPL for 24 h, cells were harvested and washed with PBS. The cells were resuspended with 500 μL PI binding buffer (MultiSciences Co., China, CCS012) and incubated in dark for 30 min. Cell cycle was analyzed by flow cytometer (Beckman Coulter CytoFLEXLX).

### Co-culture of GC cells and CAFs

Co-culture model was established using 6 wells type transwell plates (Corning Co., Cat.3450) to clarify the effect of CAFs on MKN45 or BGC-823. Briefly, CAFs (1 × 10^5^) were seeded on upper chamber, and MKN45 or BGC-823 (2.0 × 10^5^) were seeded into the lower chamber of 6 wells. On the next day, the insert-loading cells were placed into the upper compartment of the same 6-well type co-culture system and incubated for 24 h. For the apoptosis assay, the co-culture system was treated with SN38 (1 μM), TPL (12.5 nM) or SN38 (1 μM) plus TPL (12.5 nM) for another 24 h. After treatments, GC cells were harvested and washed with PBS for Annexin V-PI cell apoptosis assay (MultiSciences Co., China, AP101) using flow cytometry.

### Quantitative real-time PCR

Total RNA (1 ug) was extracted from CAFs which treated with 12.5 nM or 25 nM TPL using Trizol reagent (Invitrogen, Camarillo, CA, USA) following the manufacturer’s instructions and reverse transcribed into cDNA with PrimeScript™ RT reagent Kit (Takara, Kusatsu, Japan). qPCR was then performed using SYBR Premix Ex Taq (Takara) on a LightCycler 480 (Roche, Mannheim, Germany) PCR instrument in triplicate.

### Western blot analysis

Total cellular proteins were prepared from cell lysates with lysis buffer. As for CAFs related proteins detection, cells were treated with TPL at the concentration of 12.5 nM or 25 nM for 48 h before protein extraction. As for apoptosis-related proteins detection, cells were treated with SN38 (1 μM), TPL (12.5 nM) or SN38 (1 μM) plus TPL (12.5 nM) for 24 h with before protein extraction. Co-cultured of GC cells and CAFs were conducted as described above. Then the cells were treated with SN38 (1 μM), TPL (12.5 nM) or SN38 (1 μM) plus TPL (12.5 nM) for another 24 h. After the protein concentration of each sample was adjusted, SDS–polyacrylamide gel electrophoresis was performed to separate proteins. Subsequently, the protein bands were transferred to a polyvinylidene fluoride (PVDF) membrane. The specific primary antibodies were used as follows: FAP (Abcam, ab207178, 1:1000), α-SMA (Sigma, A2547, 1:1000), PARP (Proteintech, 66,520, 1:5000), Caspase 3 (Proteintech, 19,677, 1:1000), Bax (Proteintech, 60,267, 1:5000), Cyclin B1 (CST, #4318, 1:1000), Cyclin D1 (CST, #2978, 1:1000), Phospho-NF-κB p65 (Ser536) (CST, #3033, 1:1000), NF-κB p65 (CST, #8242, 1:1000), GAPDH (CST, #97,166, 1:1000). The level of target proteins was detected using the Syngene GeneGenius gel imaging system (Syngene, Cambridge, UK).

### Subcutaneous and intraperitoneal tumor models

6–8 weeks old female Balb/c athymic nude mice were purchased from Vital River (Beijing, China). The subcutaneous GC MKN45 tumor models were established by injecting 5 × 10^6^ MKN45 cells in the left flank and 5 × 10^6^ MKN45 plus 2.5 × 10^6^ CAFs cells in right flank (n = 3) (Additional file 2: Fig. S8a). The tumor volume was evaluated by measuring the length (L) and width (W) with a caliper and calculated using the following formula: V = (L × W^2^)/2, with W smaller than L. The CAFs-containing intraperitoneal tumor model was generated by i. p. injecting 5 × 10^6^ BGC-823-luci with 2.5 × 10^6^ CAFs cells suspended in 200 μL PBS. The intraperitoneal tumor was measured via the IVIS (PerkinElmer IVIS Lumina XRMS Series III imaging system) by in vivo luciferase BLI. The 15 mg/mL D-luciferin in 100 μL PBS was i. p. injected and after 10 min the mice were imaged and the tumor photometry was analyzed in Living Image 3.1.0. All animal studies were approved by the Animal Care and Use Committee of the Second Affiliated Hospital of Zhejiang University School of Medicine and designed according to the guidelines for the care and use of laboratory animals.

### Antitumor study in CAFs-containing intraperitoneal tumor model

The CAFs-containing intraperitoneal tumor models were established as described above. The tumor-bearing mice were randomly divided into 5 groups (n = 4). One weeks after intraperitoneal inoculation of the BGC-823-luci cells plus CAFs on mice, the antitumor therapy was started. The mice were tail-vein injected with one of the following formulations: PBS, PSN38, TPL-nsa, PSN38 + TPL-nsa, PSN38@TPL-nsa. The SN38 and TPL equivalent dose at 10 mg/kg and 0.3 mg/kg respective was used. The treatment was initiated on day 0, followed by 3 repeated injections once every 2 days. The mice were observed by BLI at day 0, 6, 12 (Fig. 5a). On the 12th day after BLI, the tumor-bearing mice were sacrificed and the intraperitoneal metastatic tumor was resected, counted and weighed.

### Antitumor study in GC PDX tumor

The protocol of construction of GC PDX models are described in Additional file 1. In vivo antitumor efficiency evaluation was performed in the P1418F4 PDX. When the tumor volume reached ≈ 50 mm^3^, the mice were randomized into 5 groups (n = 8). PBS, PSN38, TPL-nsa, PSN38 + TPL-nsa or PSN38@TPL-nsa was intravenously injected via the tail vein every 2 days for a total of 3 injections at a SN38 concentration of 10 mg/kg and TPL concentration of 0.3 mg/kg (Fig. 6a). The length and width of the tumors were recorded individually as described above, as well as the body weights. At the end of the experiment, mice were sacrificed in a humanitarian way and tumor tissues were resected, weighed and divided into two parts, one for immunofluorescence staining and the other one was fixed in 4% Paraformaldehyde (PFA) for paraffin embedding. Meanwhile, blood samples were collected and subjected for whole blood count, renal and liver function tests.

### Histochemical and immunofluorescence assays

The paraffin-embedded tissues were sectioned into 4 μm slices and stained with hematoxylin and eosin (H&E, Sigma), Ki67 (Proteintech) or MASSON Trichrome Stain Kit (Servicebio Co) following manufacturer's instructions. Immunofluorescence staining was also performed as recommended by the manufacturer. Briefly, the paraffin-embedded tumor sections with a thickness of 4 μm were dewaxed and antigen repaired, and blocked with 3% BSA for 60 min at room temperature and then incubated with the primary antibody for FAP (Abcam, ab207178, 1:200), α-SMA (Sigma, A2547, 1:200) at 4℃ overnight. The sections were rinsed 3 times with PBST (PBS containing 0.5% Triton x-100) and further incubated with Alexa Fluor 488 labeled second antibody (Invitrogen Co., A32731, 1:200) or Alexa Fluor 647 labeled second antibody (Invitrogen Co., A32728, 1:200) for 1 h at room temperature in the dark. Slides were rinsed 3 times with PBST and nuclei were stained with DAPI (FUDE Co., FD9637) staining buffer for 15 min. Fluorescent images were acquired by a fluorescence microscope (Leica DM6B).

### Statistical analysis

All experiments were performed independently in triplicate at least. Results are shown as mean ± SD. The Kaplan–Meier curve was drawn and the log-rank test was used to test the significant difference of overall survival among the groups. Statistical analysis was performed using the two-tailed unpaired Student's t-test by GraphPad Prism software (version 6.0.) The value of *p* < 0.05 was considered statistically significant.

## Supplementary Information

**Additional file 1.** Additional materials and methods.

**Additional file 2: Figure S1.** Synthetic schemes of compounds involved in this work. **Figure S2.** FT-IR spectra of PEG_5K_ and PSN38. **Figure S3.** ^1^H-NMR spectrum of various compounds synthesized. **Figure S4.** Isolation and identification of CAFs from gastric cancer (GC) tissues. **Figure S5.** Western blotting assays of FAP and α-SMA in GC cells (MKN45, SGC7901, AGS, BGC-823), normal gastric epithelial cells (GES-1), CAFs and corresponding NAFs. **Figure S6.** Immortalization of CAFs. **Figure S7.** CAFs promoted proliferation and migration of GC cells. **Figure S8.** The role of CAFs in GC tumor formation and metastasis. **Figure S9.** **a** MKN45, BGC-823 and CAFs were treated with different concentrations of TPL for 48 h, and the cell viability was assessed by CCK-8 assay. **b** Western Blot assay of caspase-3, PARP and BAX family proteins of MKN45, BGC-823 and CAFs treated with TPL, SN38 or a combination of TPL and SN38 for 24 h. **Figure S10.** In vivo safety evaluation of different therapies. **Table S1.** Examples of nanoparticle-mediated combination therapies for cancer treatment in mice.

## Data Availability

All data generated or analysed during this study are included in this published article.

## References

[1] Smyth EC, Nilsson M, Grabsch HI (2020). Gastric cancer. Lancet.

[2] Sung H, Ferlay J, Siegel RL (2021). Global cancer statistics 2020: GLOBOCAN estimates of incidence and mortality worldwide for 36 cancers in 185 countries. CA Cancer J Clin.

[3] Ajani JA, Rodriguez W, Bodoky G (2010). Multicenter phase III comparison of cisplatin/S-1 with cisplatin/infusional fluorouracil in advanced gastric or gastroesophageal adenocarcinoma study: the FLAGS trial. J Clin Oncol.

[4] Yamada Y, Higuchi K, Nishikawa K (2015). Phase III study comparing oxaliplatin plus S-1 with cisplatin plus S-1 in chemotherapy-naive patients with advanced gastric cancer. Ann Oncol.

[5] Zhang Z, Cai M, Bao C (2019). Endoscopic Cerenkov luminescence imaging and image-guided tumor resection on hepatocellular carcinoma-bearing mouse models. Nanomedicine.

[6] Chia NY, Tan P (2016). Molecular classification of gastric cancer. Ann Oncol.

[7] Bejarano L, Jordao MJC, Joyce JA (2021). Therapeutic targeting of the tumor microenvironment. Cancer Discov.

[8] Sahai E, Astsaturov I, Cukierman E (2020). A framework for advancing our understanding of cancer-associated fibroblasts. Nat Rev Cancer.

[9] Ham IH, Oh HJ, Jin H (2019). Targeting interleukin-6 as a strategy to overcome stroma-induced resistance to chemotherapy in gastric cancer. Mol Cancer.

[10] Zhai J, Shen J, Xie G (2019). Cancer-associated fibroblasts-derived IL-8 mediates resistance to cisplatin in human gastric cancer. Cancer Lett.

[11] Uchihara T, Miyake K, Yonemura A (2020). Extracellular vesicles from cancer-associated fibroblasts containing annexin A6 Induces FAK-YAP activation by stabilizing beta1 integrin, enhancing drug resistance. Cancer Res.

[12] Bang YJ, Van Cutsem E, Feyereislova A (2010). Trastuzumab in combination with chemotherapy versus chemotherapy alone for treatment of HER2-positive advanced gastric or gastro-oesophageal junction cancer (ToGA): a phase 3, open-label, randomised controlled trial. Lancet.

[13] Cancer Genome Atlas Research N (2014). Comprehensive molecular characterization of gastric adenocarcinoma. Nature.

[14] Chen X, Song E (2019). Turning foes to friends: targeting cancer-associated fibroblasts. Nat Rev Drug Discov.

[15] Dauer P, Zhao X, Gupta VK (2018). Inactivation of cancer-associated-fibroblasts disrupts oncogenic signaling in pancreatic cancer cells and promotes its regression. Cancer Res.

[16] Banerjee S, Modi S, McGinn O (2016). Impaired synthesis of stromal components in response to minnelide improves vascular function, drug delivery, and survival in pancreatic cancer. Clin Cancer Res.

[17] Xu H, Liu B (2019). Triptolide-targeted delivery methods. Eur J Med Chem.

[18] Ma L, Kohli M, Smith A (2013). Nanoparticles for combination drug therapy. ACS Nano.

[19] Li C, Dong D, Li L (2020). Classification of severe and critical Covid-19 using deep learning and radiomics. Ieee J Biomed Health.

[20] Ahmad N, Bhatnagar S, Ali SS (2015). Phytofabrication of bioinduced silver nanoparticles for biomedical applications. Int J Nanomedicine.

[21] Khatoon A, Khan F, Ahmad N (2018). Silver nanoparticles from leaf extract of Mentha piperita: eco-friendly synthesis and effect on acetylcholinesterase activity. Life Sci.

[22] Abdelhamid HN, Dowaidar M, Langel U (2020). Carbonized chitosan encapsulated hierarchical porous zeolitic imidazolate frameworks nanoparticles for gene delivery. Micropor Mesopor Mat..

[23] Jiang TY, Sun WJ, Zhu QW (2015). Furin-mediated sequential delivery of anticancer cytokine and small-molecule drug shuttled by graphene. Adv Mater.

[24] He QJ, Gao Y, Zhang LX (2011). A pH-responsive mesoporous silica nanoparticles-based multi-drug delivery system for overcoming multi-drug resistance. Biomaterials.

[25] Abdelhamid HN, Dowaidar M, Hallbrink M (2020). Gene delivery using cell penetrating peptides-zeolitic imidazolate frameworks. Micropor Mesopor Mat..

[26] Ahmad N, Bhatnagar S, Saxena R (2017). Biosynthesis and characterization of gold nanoparticles: Kinetics, in vitro and in vivo study. Mater Sci Eng C Mater Biol Appl.

[27] Xu S, Ling S, Shan Q (2021). Self-activated cascade-responsive sorafenib and USP22 shRNA co-delivery system for synergetic hepatocellular carcinoma therapy. Adv Sci (Weinh).

[28] Han L, Huang R, Li J (2011). Plasmid pORF-hTRAIL and doxorubicin co-delivery targeting to tumor using peptide-conjugated polyamidoamine dendrimer. Biomaterials.

[29] Tardi PG, Dos Santos N, Harasym TO (2009). Drug ratio-dependent antitumor activity of irinotecan and cisplatin combinations in vitro and in vivo. Mol Cancer Ther.

[30] Ahlawat J, Barroso GG, Asil SM (2020). Nanocarriers as potential drug delivery candidates for overcoming the blood-brain barrier: challenges and possibilities. ACS Omega.

[31] Sahai N, Gogoi M, Ahmad N (2021). Mathematical modeling and simulations for developing nanoparticle-based cancer drug delivery systems: a review. Curr Pathobiol Rep.

[32] Lancet JE, Cortes JE, Hogge DE (2014). Phase 2 trial of CPX-351, a fixed 5:1 molar ratio of cytarabine/daunorubicin, vs cytarabine/daunorubicin in older adults with untreated AML. Blood.

[33] Noel P, Von Hoff DD, Saluja AK (2019). Triptolide and its derivatives as cancer therapies. Trends Pharmacol Sci.

[34] Liu X, Wang J, Shen Y (2015). Amphiphilic block copolymer of SN38 prodrugs by atom transfer radical polymerization: synthesis, kinetic studies and self-assembly. J Control Release.

[35] Wang L, Liu X, Zhou Q (2017). Terminating the criminal collaboration in pancreatic cancer: nanoparticle-based synergistic therapy for overcoming fibroblast-induced drug resistance. Biomaterials.

[36] Kong C, Li Y, Liu Z (2019). Targeting the oncogene KRAS mutant pancreatic cancer by synergistic blocking of lysosomal acidification and rapid drug release. ACS Nano.

[37] Dong H, Pang L, Cong H (2019). Application and design of esterase-responsive nanoparticles for cancer therapy. Drug Deliv.

[38] Biffi G, Tuveson DA (2021). Diversity and biology of cancer-associated fibroblasts. Physiol Rev.

[39] Erez N, Truitt M, Olson P (2010). Cancer-associated fibroblasts are activated in incipient neoplasia to orchestrate tumor-promoting inflammation in an NF-kappaB-dependent manner. Cancer Cell.

[40] Xu Y, Villalona-Calero MA (2002). Irinotecan: mechanisms of tumor resistance and novel strategies for modulating its activity. Ann Oncol.

[41] Byrne AT, Alferez DG, Amant F (2017). Interrogating open issues in cancer precision medicine with patient-derived xenografts. Nat Rev Cancer.

[42] Feig C, Gopinathan A, Neesse A (2012). The pancreas cancer microenvironment. Clin Cancer Res.

[43] Glimelius B, Ekstrom K, Hoffman K (1997). Randomized comparison between chemotherapy plus best supportive care with best supportive care in advanced gastric cancer. Ann Oncol.

[44] Wagner AD, Syn NL, Moehler M (2017). Chemotherapy for advanced gastric cancer. Cochrane Database Syst Rev.

[45] Xu W, Yang Z, Lu N (2016). Molecular targeted therapy for the treatment of gastric cancer. J Exp Clin Cancer Res.

[46] Ding N, Xu S, Zheng S (2021). "Sweet tooth"-oriented SN38 prodrug delivery nanoplatform for targeted gastric cancer therapy. J Mater Chem B.

[47] Liu M, Zheng S, Zhang X (2018). Cerenkov luminescence imaging on evaluation of early response to chemotherapy of drug-resistant gastric cancer. Nanomedicine.

[48] Guo H, Yu J, Hu Z (2018). A hybrid clustering algorithm for multiple-source resolving in bioluminescence tomography. J Biophotonics.

[49] Dowaidar M, Nasser Abdelhamid H, Hallbrink M (2018). Chitosan enhances gene delivery of oligonucleotide complexes with magnetic nanoparticles-cell-penetrating peptide. J Biomater Appl.

[50] Ozdemir BC, Pentcheva-Hoang T, Carstens JL (2014). Depletion of carcinoma-associated fibroblasts and fibrosis induces immunosuppression and accelerates pancreas cancer with reduced survival. Cancer Cell.

[51] Kam PC, Liew S (2002). Traditional Chinese herbal medicine and anaesthesia. Anaesthesia.

[52] Yu H, Lin L, Zhang Z (2020). Targeting NF-kappaB pathway for the therapy of diseases: mechanism and clinical study. Signal Transduct Target Ther.

[53] Su S, Chen J, Yao H (2018). CD10(+)GPR77(+) cancer-associated fibroblasts promote cancer formation and chemoresistance by sustaining cancer stemness. Cell.

[54] Sun Y, Campisi J, Higano C (2012). Treatment-induced damage to the tumor microenvironment promotes prostate cancer therapy resistance through WNT16B. Nat Med.

[55] Tentler JJ, Tan AC, Weekes CD (2012). Patient-derived tumour xenografts as models for oncology drug development. Nat Rev Clin Oncol.

[56] Hu C, Wang Z, Zhai L (2013). Effects of cancer-associated fibroblasts on the migration and invasion abilities of SGC-7901 gastric cancer cells. Oncol Lett.

