# Supplementary material for: Prodrug polymeric micelles integrating cancer-associated fibroblasts deactivation and synergistic chemotherapy for gastric cancer
Source: J Nanobiotechnology. 2021 Nov 21;19:381. doi: 10.1186/s12951-021-01127-5 (PMC8607732; doi:10.1186/s12951-021-01127-5)
Supplement: Supplementary file 1 — Additional file 1. Additional materials and methods. [file 12951_2021_1127_MOESM1_ESM.docx]

**Additional Materials and Methods**

**Prodrug polymeric micelles integrating** **cancer-associated fibroblasts deactivation and synergistic chemotherapy for gastric cancer**

Sheng Zheng^1,3,4,#^, Jiafeng Wang^2,4,#^, Ning Ding^1,3,4^, Wenwen Chen^1,3,4^, Hongda Chen^2,4^, Meng Xue^1,3,4^, Fei Chen^1,3,4^, Jiaojiao Ni^1,3,4^, Zhuo Wang^1,3,4^, Zhenghua Lin^1,3,4^, Haiping Jiang^5^, Xiangrui Liu^1,2,4,*^, Liangjing Wang^1,3,4,^^*^

^1^Department of Gastroenterology, The Second Affiliated Hospital of Zhejiang University School of Medicine, 88 Jiefang Road, Hangzhou, 310009, Zhejiang, China

^2^Department of Pharmacology, Zhejiang University School of Medicine, Hangzhou 310058, China

^3^Institute of Gastroenterology, Zhejiang University, Hangzhou 310058, China

^4^Cancer Center, Zhejiang University, Hangzhou 310058, China

^5^Department of Medical Oncology, The First Affiliated Hospital of Medical School of Zhejiang University, Hangzhou, 310016, China

*Correspondence: [wangljzju@zju.edu.cn](mailto:wangljzju@zju.edu.cn), xiangrui@zju.edu.cn

^#^Sheng Zheng and Jiafeng Wang contributed equally to this work

**1. Synthesis of PEG_5k_-P(MMESSN38)_5k_ (PSN38)**

1.1 Synthesis of 4-Cyano-4-(2-phenylethanesulfanylthiocarbonyl) sulfanylpentanoic acid (PETTC) (Fig. S1a)

Sodium hydride (60% in oil) (3.15 g, 79 mmol) was dissolved in 150 ml diethyl ether and then 2-Phenylethanethiol (10.5 g, 76 mmol) was added dropwise into the solution at 5~10 ℃. The reaction solution was stirred for 1 h and then CS_2_ (6.0 g, 79 mmol) was added dropwise at 0 ℃ to generate a yellow precipitate. After vigorous stirring for 0.5 h, the yellow precipitate was collected via vacuum filtration, washed with diethyl ether and dried to afford the intermediate product without further purification. 7.5 g product from previous steps was suspended in 100 ml diethyl ether and solid iodine (7.5 g, 29 mmol) was added with vigorous stirring for 1 h at room temperature. The precipitate NaI was filtrated to obtain a brown filtrate which was washed with saturated sodium thiosulfate aqueous solution (50 ml, 3 times) and brine (50 ml, 3 times). The product was collected via rotary evaporation without further purification. 4,4′-azobis(4-cyanopentanoicacid) (ACVA) (11.3 g, 40 mmol) and the product from the previous step (11.3 g) was dissolved in 100 ml anhydrous ethyl acetate (100 ml). Then the solution was bubbled with dry N_2_ for 30 min and subsequently was heated reflux at 80 ℃ for 18 h. The crude product of PETTC was obtained by washing the reaction solution with water (50 ml, 3 times) and brine (50 ml, 3 times). The crude product was further purified by column chromatography (18.8 g, 72.1%) (Fig. S3a). ^1^H NMR (400 MHz, CDCl_3_) δ (ppm): δ 7.36 – 7.21 (m, 5H), 3.64 – 3.49 (m, 2H), 3.07 – 2.92 (m, 2H), 2.79 – 2.63 (m, 2H), 2.60 – 2.48 (m, 1H), 2.43 – 2.33 (m, 1H), 1.88 (s, 3H).

- 1. Synthesis of PEG_5k_-PETTC (Fig. S1b)

Anhydrous poly (ethylene glycol) methyl ether (Mn = 5,000 Da) (5 g, 1 mmol), PETTC (1.4 g, 4 mmol) and 4-Dimethylaminopyridine (DMAP, 0.2 g, 1.6 mmol) were dissolved in 100 ml anhydrous Dichloromethane (DCM). Dicyclohexylcarbodiimide (DCC, 1.65 g, 8 mmol) dissolved in 50 ml anhydrous DCM was added dropwise into the reaction solution at 0 ℃. The reaction was then stirred for 3 days at room temperature. The insoluble dicyclohexylurea (DCU) impurity was removed by filtration and the filtrate was concentrated via rotary evaporation. Further purification was affected by reprecipitation from cold anhydrous ethanol for 3 times to afford a faint yellow powder PEG_5k_-PETTC (5 g, 94.7%) (Fig. S3b).

- 1. Synthesis of MMESSN38 (Fig. S1c)

7-ethyl-10-hydroxycamptothecin (SN38, 1 g, 2.5 mmol), mono-2-(methacryloyloxy) ethyl succinate (2.4 g, 10 mmol) and 10 ml pyridine were suspended in 100 ml anhydrous DCM. 40 ml anhydrous DCM containing (3-dimethylaminopropyl)-3-ethylcarbodiimide hydrochloride (EDC·HCl, 1.9 g, 10 mmol) was added dropwise to the reaction solution at 0 ℃. After stirring overnight, the reaction solution was washed with 1M HCl (50 ml, 3 times) and dried by anhydrous NaSO_4_. Crude product was obtained by reprecipitation in cold ethyl ether and further purified by column chromatography (1.3 g, 86%) (Fig. S3c). ^1^H NMR (400 MHz, CDCl_3_) δ (ppm): δ 8.22 (d, J = 9.1 Hz, 1H), 7.81 (d, J = 2.5 Hz, 1H), 7.67 (s, 1H), 7.54 (dd, J = 9.1, 2.5 Hz, 1H), 6.13 (s, 1H), 5.73 (d, J = 16.3 Hz, 1H), 5.58 (p, J = 1.7 Hz, 1H), 5.37 – 5.18 (m, 3H), 4.51 – 4.33 (m, 4H), 4.18 (s, 1H), 3.14 (q, J = 7.6 Hz, 2H), 3.00 (dd, J = 7.6, 5.7 Hz, 2H), 2.85 (dd, J = 7.5, 5.7 Hz, 2H), 1.98 – 1.85 (m, 5H), 1.40 (t, J = 7.6 Hz, 3H), 1.02 (t, J = 7.3 Hz, 3H).

- 1. Synthesis of PSN38 (Fig. S1d)

PEG_5k_-PETTC (200 mg, 0.04 mmol), monomer MMESSN38 (200 mg, 0.34 mmol), and Azobisisobutyronitrile (AIBN, 2 mg, 0.012 mmol) were dissolved in anhydrous 1,4-dioxane (4 ml) and bubbled with dry N_2_ for 30 min. The reaction was stirring at 75 ℃ for 12 h. After that, the product was obtained by reprecipitation in 100 ml cold anhydrous ethanol for 3 times and dried in a vacuum oven. MMESSN38 content in polymer conjugate was analyzed by ^1^H-NMR (Fig. S3d).

**2. Synthesis of triptolide-naphthalene sulfonamide (TPL-nsa)**

Triptolide (0.36 g, 1 mmol), succinic anhydride (0.5 g, 5 mmol), DMAP (0.6 g, 5 mmol) and Trimethylamine (TEA, 1.03 g, 5 mmol) were dissolved in 2 ml anhydrous DCM and reacted for 1 h at room temperature. The reaction solution was diluted with 100 ml DCM, washed with 1 M HCl (30 ml, 3 times) and water (30 ml, 3 times) and dried by anhydrous NaSO_4_. The intermediate product was collected via rotary evaporation without further purification. Then the intermediate product (0.46 g, 1 mmol), naphthalene sulfonamide (0.25 g, 1.2 mmol), DMAP (0.18 g, 1.5 mmol) and EDC·HCl (0.23 g, 1.2 mmol) were dissolved in 20 ml anhydrous DCM and stirred overnight at room temperature. The crude product was further purified by column chromatography to yield triptolide-naphthalene sulfonamide (TPL-nsa) (Fig. S1e and Fig. S3e). ^1^H NMR (400 MHz, CDCl_3_): δ 9.46 (s, 1H), 8.67 (d, J = 1.8 Hz, 1H), 8.06 – 7.95 (m, 3H), 7.94 – 7.89 (m, 1H), 7.65 (dddd, J = 20.4, 8.2, 7.0, 1.4 Hz, 2H), 5.06 (s, 1H), 4.74 – 4.63 (m, 2H), 3.96 (d, J = 3.2 Hz, 1H), 3.64 (dd, J = 3.1, 0.9 Hz, 1H), 3.49 (d, J = 5.6 Hz, 1H), 2.88 – 2.46 (m, 5H), 2.17 (dt, J = 14.9, 5.8 Hz, 2H), 1.95 – 1.84 (m, 2H), 1.61 (t, J = 6.4 Hz, 1H), 1.25 (td, J = 12.1, 5.8 Hz, 2H), 1.04 (s, 3H), 0.89 (d, J = 7.0 Hz, 3H), 0.81 (d, J = 6.9 Hz, 3H).

**3. Isolation and culturing of fibroblasts**

Fibroblast isolation was approved by the ethics committee of The Second Affiliated Hospital of Zhejiang University School of Medicine and we got informed consent from all patients. Human gastric cancer (GC) specimens were obtained from patients that underwent tumor resection surgery at The Second Affiliated Hospital of Zhejiang University School of Medicine (Hangzhou, China). The fresh specimen from radical correction of GC was taken from the operating room and sheared into small blocks 1 mm in diameter on an aseptic bench and then placed uniformly onto the culture face of a disposable culture flask with an elbow straw (the interval between the blocks was 5 mm). High-sugar DMEM (2 ml) containing 20% fetal bovine serum (FBS) was added, the culture flask was then inverted and cultured for 4 h in the incubator containing 5% CO_2_ at 37˚C. Subsequently, the culture flask was overturned. The culture liquid was replaced on the third day and then every subsequent three days. The soonest cells were seen to appear was following 5 to 7 days. After ~2 to 3 weeks, cells covered the whole base of the flask. The enzyme digestion method was used to purify the cells. Following the first passage, the medium was replaced with DMEM medium containing 10% FBS and 1% antibiotics to conduct a conventional culture. In the current study, three paired cancer associated fibroblasts (CAFs) and normal associated fibroblasts (NAFs) samples harvested from three different patients were used. The pathologic characteristics are described below.

| **Fibroblasts** | **Gender** | **Age**  **(year)** | **Tumor size (cm)** | **Depth of invasion** | **Location** | **Operation** | **WHO**  **classification** | **Lauren**  **classification** | **Metastasis** |
| --- | --- | --- | --- | --- | --- | --- | --- | --- | --- |
| CAF1/NAF1 | Male | 55 | 3.5*2.5 | Subserous | Body | Total | Moderately-Poorly adenocarcinoma | Mixed | Lymph node |
| CAF2/NAF2 | Female | 57 | 9*6.5 | Serosa | Body | Total | Mucinous adenocarcinoma | Mixed | Lymph node |
| CAF3/NAF3 | Male | 55 | 3*2 | Submucosa | Body | Total | Poorly adenocarcinoma | Diffuse | NA |

**4. Construction of GC patient-derived xenografts model**

Fresh tumor tissue was obtained from GC patients who under surgical resection and didn’t have any chemotherapeutic or locoregional treatments before the surgery. Resected tumors were sliced into ≈1 mm^3^ wads and then subcutaneously implanted into flanks of 5-weeks old female Balb/c athymic nude. The mice implanted with original patient’s tumor, were defined as the Founder 0 (F0). The second generation of patient-derived xenografts (PDX) model was defined as F1. We randomly selected a PDX model that originated from patient 1418 (P1418) from the PDX sample library for in vivo antitumor efficiency assessment. The pathologic characteristics are described below.

| **Patient** | **Founder** | **Gender** | **Age**  **(year)** | **Tumor size (cm)** | **Depth of invasion** | **Location** | **Operation** | **WHO**  **classification** | **Lauren**  **classification** | **Metastasis** |
| --- | --- | --- | --- | --- | --- | --- | --- | --- | --- | --- |
| P1418 | F4 | Male | 51 | 6.3*5.0 | Subserous layer | Body | Total | Moderately tubular adenocarcinoma | Mixed | Lymph node |
