# Supplementary material for: Prodrug polymeric micelles integrating cancer-associated fibroblasts deactivation and synergistic chemotherapy for gastric cancer
Source: J Nanobiotechnology. 2021 Nov 21;19:381. doi: 10.1186/s12951-021-01127-5 (PMC8607732; doi:10.1186/s12951-021-01127-5)
Supplement: Supplementary file 2 — Additional file 2: Figure S1. Synthetic schemes of compounds involved in this work. Figure S2. FT-IR spectra of PEG5K and PSN38. Figure S3. 1H-NMR spectrum of various compounds synthesized. Figure S4. Isolation and identification of CAFs from gastric cancer (GC) tissues. Figure S5. Western blotting assays of FAP and α-SMA in GC cells (MKN45, SGC7901, AGS, BGC-823), normal gastric epithelial cells (GES-1), CAFs and corresponding NAFs. Figure S6. Immortalization of CAFs. Figure S7. CAFs promoted proliferation and migration of GC cells. Figure S8. The role of CAFs in GC tumor formation and metastasis. Figure S9. a MKN45, BGC-823 and CAFs were treated with different concentrations of TPL for 48 h, and the cell viability was assessed by CCK-8 assay. b Western Blot assay of caspase-3, PARP and BAX family proteins of MKN45, BGC-823 and CAFs treated with TPL, SN38 or a combination of TPL and SN38 for 24 h. Figure S10. In vivo safety evaluation of different therapies. Table S1. Examples of nanoparticle-mediated combination therapies for cancer treatment in mice. [file 12951_2021_1127_MOESM2_ESM.docx]

**Additional Results**

**Prodrug polymeric micelles integrating** **cancer-associated fibroblasts deactivation and synergistic chemotherapy for gastric cancer**

Sheng Zheng^1,3,4,#^, Jiafeng Wang^2,4,#^, Ning Ding^1,3,4^, Wenwen Chen^1,3,4^, Hongda Chen^2,4^, Meng Xue^1,3,4^, Fei Chen^1,3,4^, Jiaojiao Ni^1,3,4^, Zhuo Wang^1,3,4^, Zhenghua Lin^1,3,4^, Haiping Jiang^5^, Xiangrui Liu^1,2,4,*^, Liangjing Wang^1,3,4,^^*^

^1^Department of Gastroenterology, The Second Affiliated Hospital of Zhejiang University School of Medicine, 88 Jiefang Road, Hangzhou, 310009, Zhejiang, China

^2^Department of Pharmacology, Zhejiang University School of Medicine, Hangzhou 310058, China

^3^Institute of Gastroenterology, Zhejiang University, Hangzhou 310058, China

^4^Cancer Center, Zhejiang University, Hangzhou 310058, China

^5^Department of Medical Oncology, The First Affiliated Hospital of Medical School of Zhejiang University, Hangzhou, 310016, China

*Correspondence: [wangljzju@zju.edu.cn](mailto:wangljzju@zju.edu.cn), xiangrui@zju.edu.cn

^#^Sheng Zheng and Jiafeng Wang contributed equally to this work


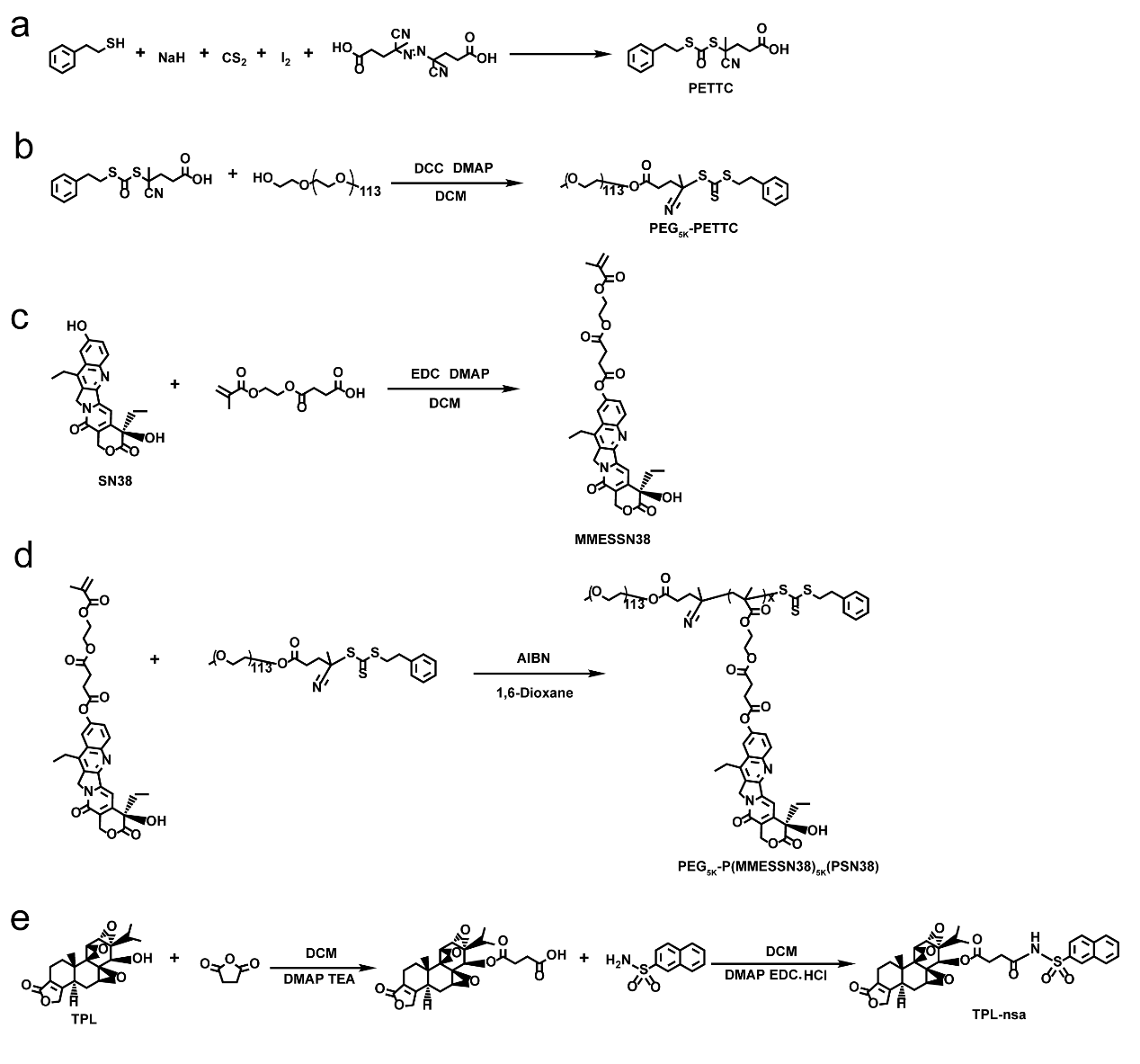


**Figure S1.** Synthetic schemes of compounds involved in this work. **a** Synthesis of PETTC. **b** Synthesis of macro-CTA agent PEG_5K_-PETTC. **c** Synthesis of MMESSN38. **d** Synthesis of PEG_5K_-P(MMESSN38)_5K_ (PSN38). **e** Synthesis of the triptolide prodrug, triptolide-naphthalene sulfonamide (TPL-nsa).


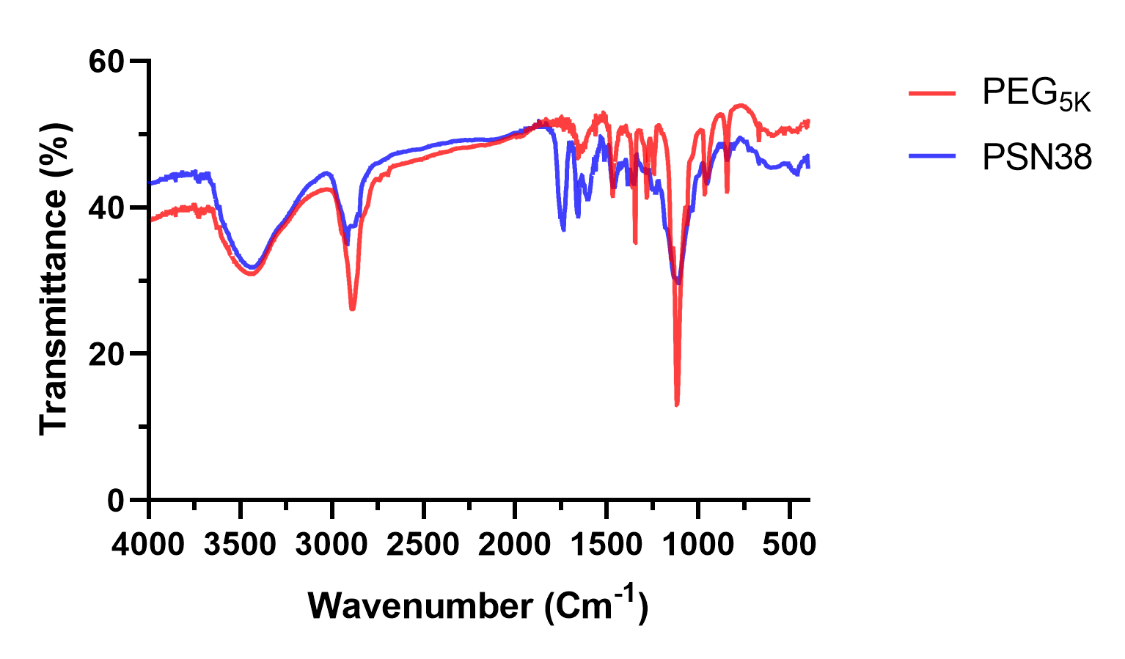


**Figure S2.** FT-IR spectra of PEG_5K_ and PSN38.


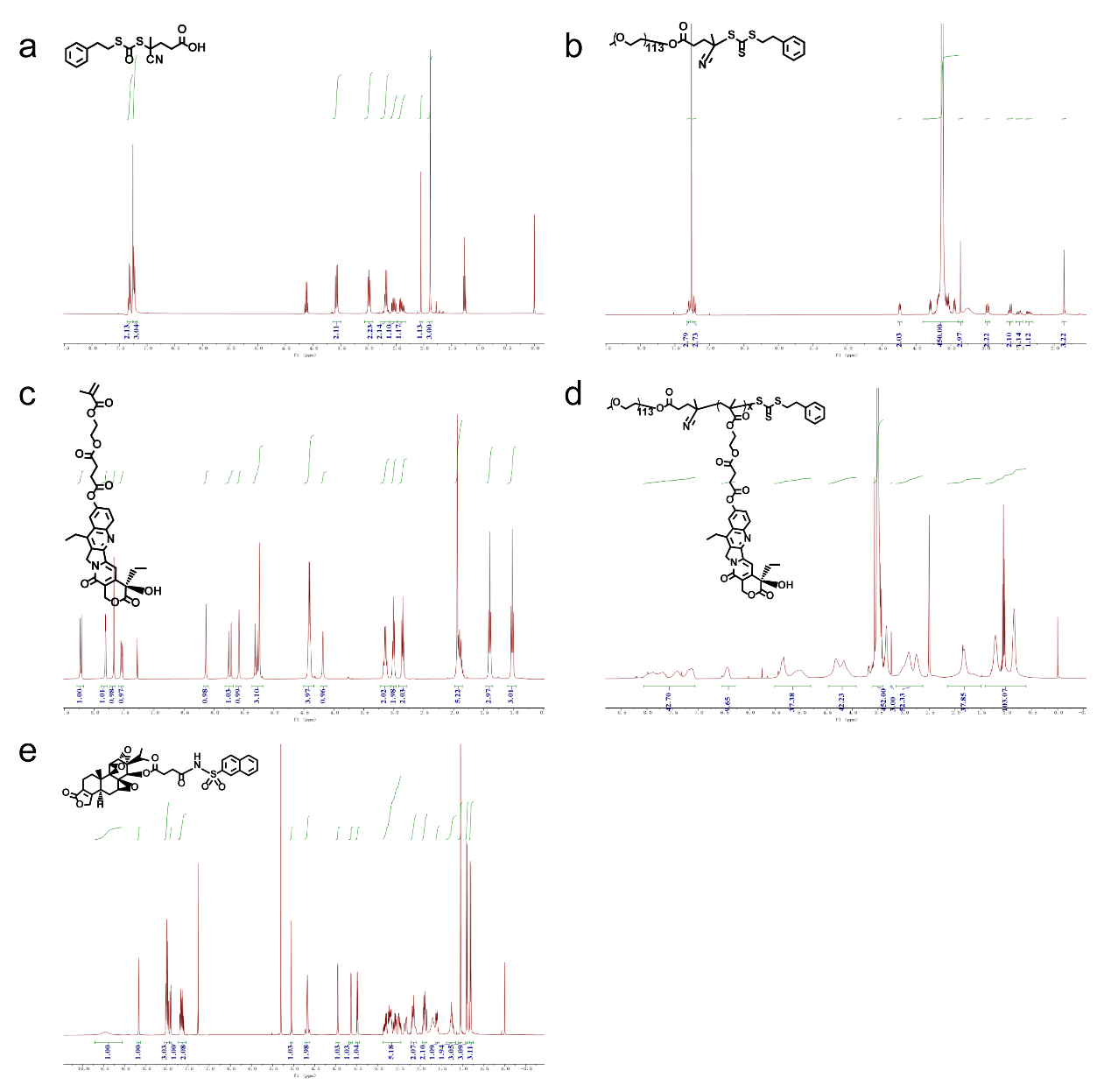


**Figure S3.** ^1^H-NMR spectrum of various compounds synthesized. **a** ^1^H-NMR spectra of PETTC in chloroform-d. **b** ^1^H-NMR spectra of PEG_5K_-PETTC in chloroform-d. **c** ^1^H-NMR spectra of MMESSN38 in chloroform-d. **d** ^1^H-NMR spectra of PSN38 in DMSO-d6. **e** ^1^H-NMR spectra of TPL-nsa in chloroform-d.


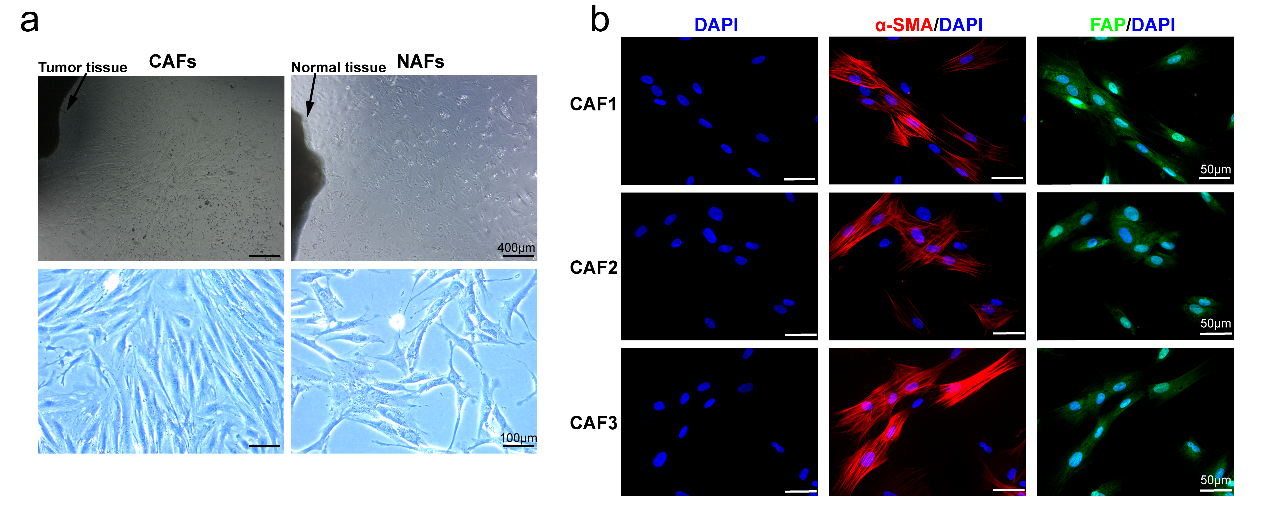


**Figure S4.** Isolation and identification of CAFs from gastric cancer (GC) tissues. **a** CAFs and NAFs were isolated from fresh GC specimens and corresponding normal stomach tissues, respectively. **b** Immunoﬂuorescence staining of α‑SMA (red) and FAP (green) in CAFs. Cell nuclei were counterstained with DAPI (blue).


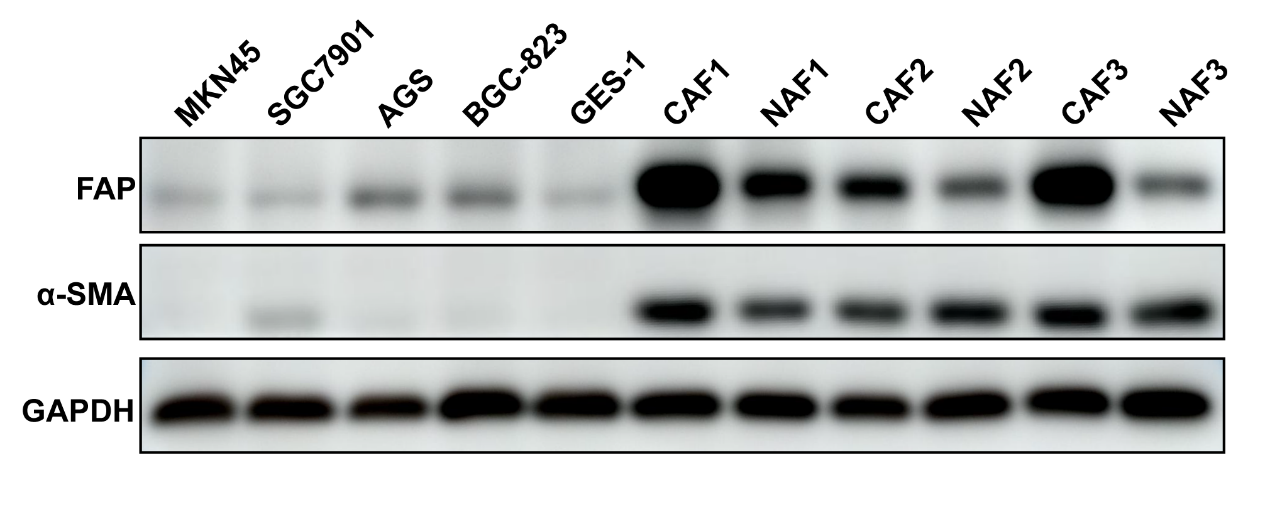


**Figure S5.** Western blotting assays of FAP and α-SMA in GC cells (MKN45, SGC7901, AGS, BGC-823), normal gastric epithelial cells (GES-1), CAFs and corresponding NAFs.


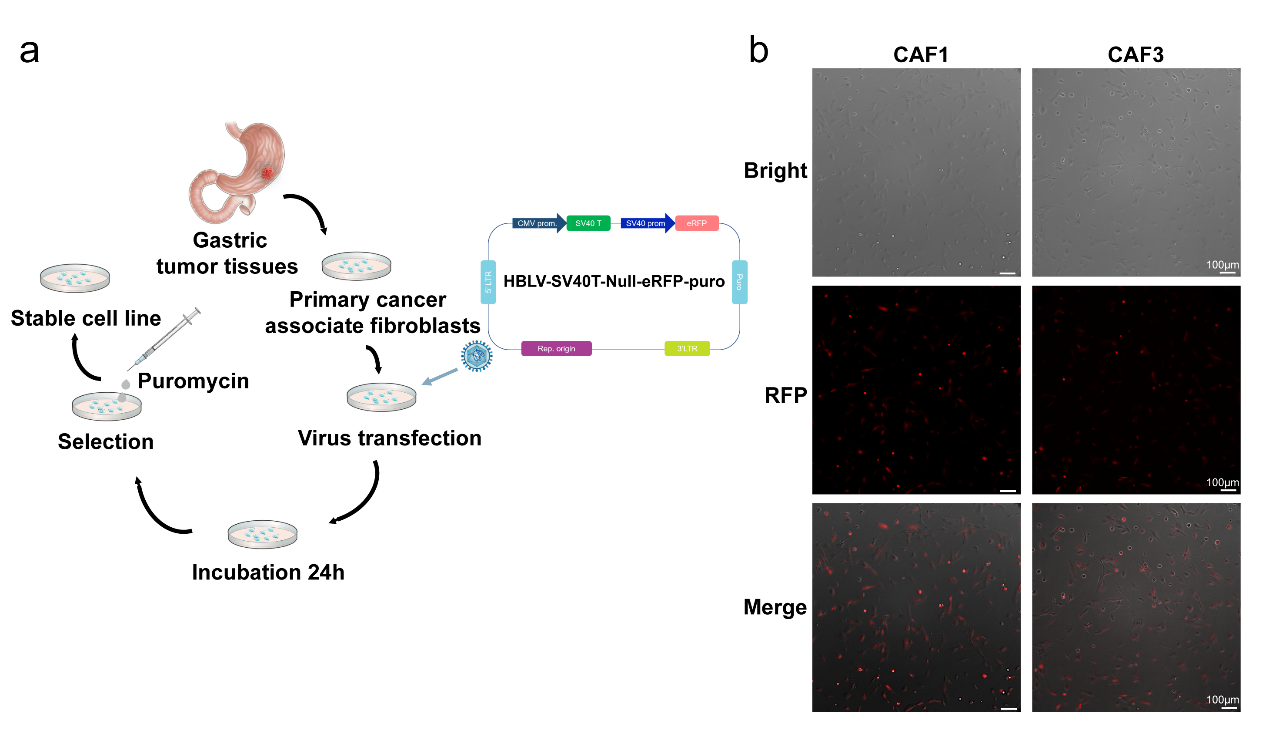


**Figure S6.** Immortalization of CAFs. **a** Flow diagram of immortalization of CAFs. Primary CAFs were transfected with lentiviral SV40-T. **b** Red fluorescent protein (RFP) was sustainably expressed in immortalization CAFs.


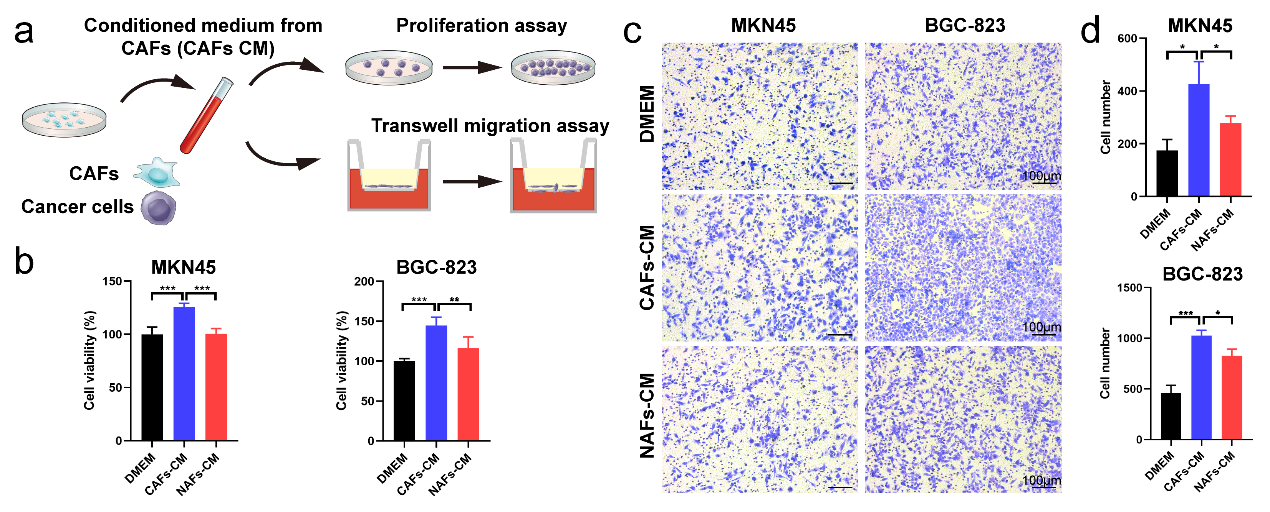


**Figure S7.** CAFs promoted proliferation and migration of GC cells. **a** The diagram of GC cells incubated with conditioned medium (CM) derived from CAFs. **b, c, d** Proliferation (b) and migration (c, d) of MKN45 and BGC-823 incubated with CM derived from CAFs or NAFs. All data are presented as mean ± SD. Unpaired Student’s t-test was used to analyze the data (**p* < 0.05; ***p* < 0.01; ****p* < 0.001).


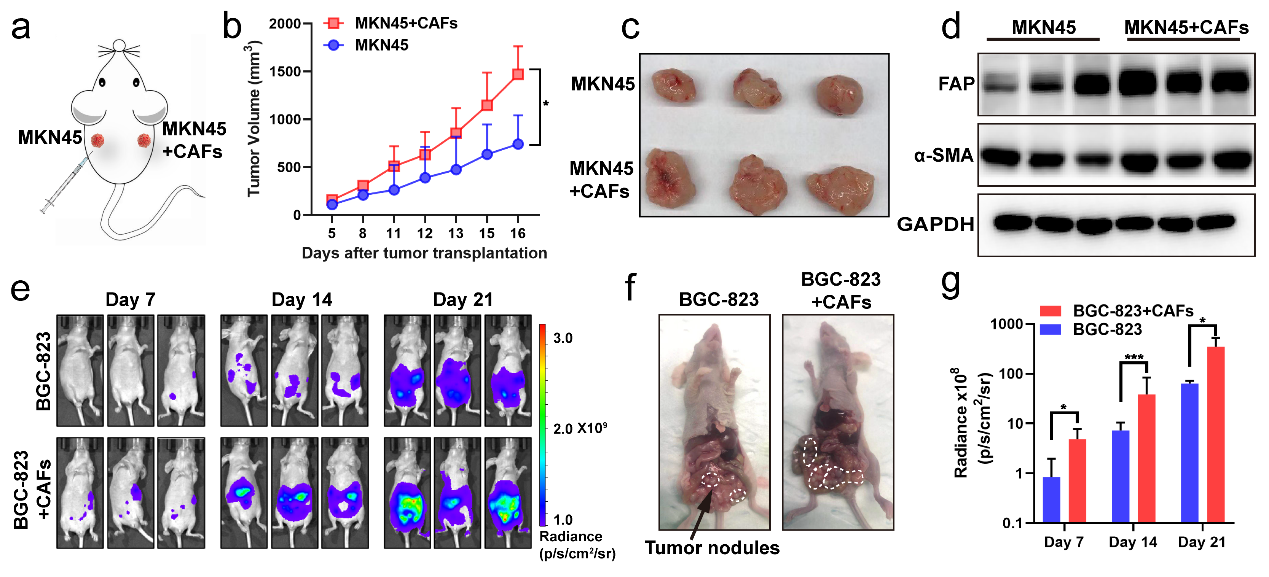


**Figure S8.** The role of CAFs in GC tumor formation and metastasis. **a** Subcutaneous GC tumor models were established by injecting MKN45 cells in the left flank and MKN45+CAFs in the right flank, respectively. **b** The tumor growth curves after cell transplantation. **c** Images of excised tumors of mice 16 days after cell transplantation. **d** Western blot assay of FAP and α-SMA of MKN45-derived tumors and MKN45+CAFs-dervied tumors *in vivo*. **e** Intraperitoneal tumor nodules development of BGC-823-luci and BGC-823-luci+CAFs was visualized via luciferase bioluminescence imaging detection. **f** Representative images of peritoneal tumor nodules distribution. **g** Quantitative analysis bioluminescence intensity of peritoneal tumor nodules. All data are presented as mean ± SD. Student’s t-test was used to analyze the data. (**p* < 0.05; ***p* < 0.01; ****p* < 0.001)


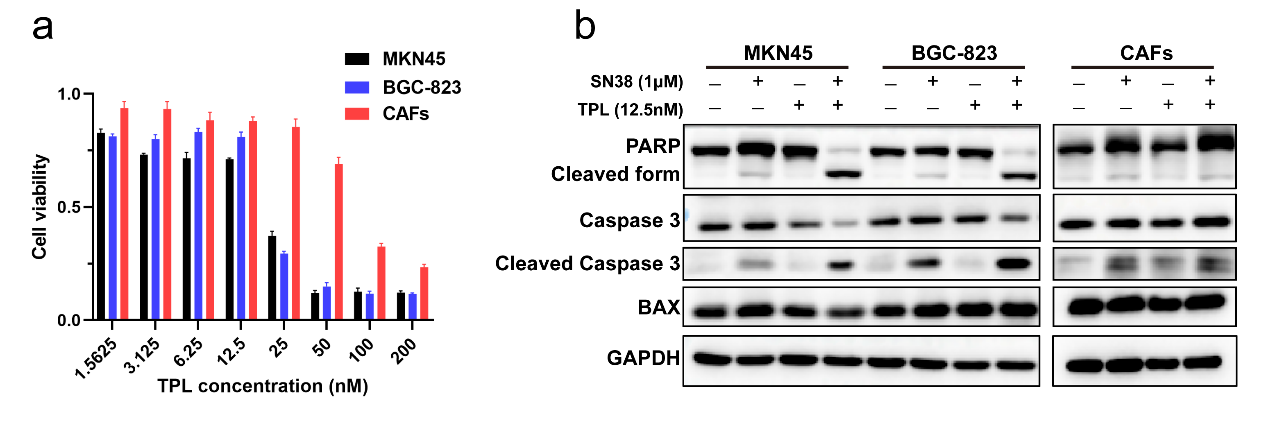


**Figure S9. a** MKN45, BGC-823 and CAFs were treated with different concentrations of TPL for 48 h, and the cell viability was assessed by CCK-8 assay. **b** Western Blot assay of caspase-3, PARP and BAX family proteins of MKN45, BGC-823 and CAFs treated with TPL, SN38 or a combination of TPL and SN38 for 24 h.


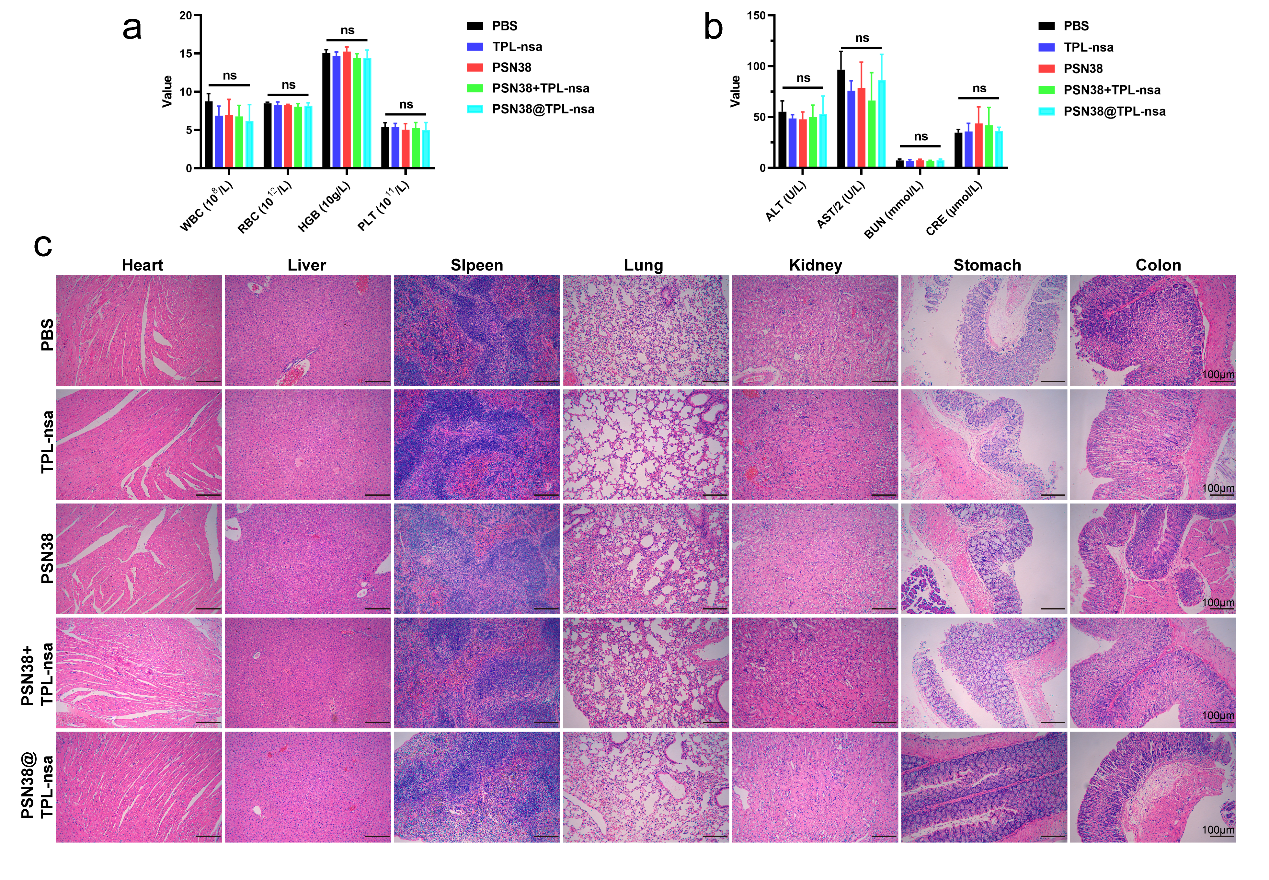


**Figure S10.** In vivo safety evaluation of different therapies. **a** Whole-cell counts of PDX model mice treated with PBS, TPL-nsa, PSN38, PSN38+TPL-nsa and PSN38@TPL-nsa. **b** Liver and kidney toxicity analysis of different therapies. The data are represented as the mean ± SD (n = 8). All the indicators were within a normal biological range. **c** Representative H&E staining of hearts, livers, spleens, lungs and kidneys excised from nude mice in the different groups (n = 8 for each group). RBC: red boold cell; HGB: hemoglobin; HCT: hematocrit; PLT: platelet count; WBC: white boold cell; NE%: neutrophil percentage; LY%: lymphocyte percentage; AST: aspartate aminotransferase; ALT: alanine aminotransferase; BUN: blood urea nitrogen; CRE: creatinine.

**Table. S1** Examples of nanoparticle-mediated combination therapies for cancer treatment in mice.

| **Nanotechnology platform** | **Active pharmaceutical ingredients** | **Therapeutic mechanism** | **Tumor model** | **Refs** |
| --- | --- | --- | --- | --- |
| **Inorganic NPs** | | | | |
| Iron oxide NPs | Doxorubicin and curcumin | Combination of chemotherapies | Glioma | [1] |
| Graphene | Doxorubicin and TRAIL | Combination of chemotherapy and cytokine-induced apoptosis | NSCLC | [2] |
| Carbon nanotube | siRNA | Combination of hyperthermia and RNAi therapy | Prostate cancer | [3] |
| Gold nanorod | Doxorubicin | Combination of hyperthermia and chemotherapy | Cervical cancer | [4] |
| **Organic NPs** | | | | |
| Polymeric micelles or NPs | SN38 and triptolide | Combination of chemotherapy and CAFs deactivation | Gastric cancer | Our study |
|  | Doxorubicin and disulfiram | Combination of chemotherapy and anti-drug resistance | Drug-resistant breast cancer | [5] |
| Liposomes or lipid-based NPs | Irinotecan and cisplatin | Combination of chemotherapies | SCLC | [6] |
| Lipid–polymer hybrid NPs | Combretastatin and doxorubicin | Combining anti-angiogenesis and chemotherapy | Melanoma and Lewis lung carcinoma | [7] |
| Dendrimers | Doxorubicin and DNA | Combination of chemotherapy and gene therapy using TRAIL-encoded plasmid | Liver cancer | [8] |

NPs, nanoparticles; NSCLC, non-small-cell lung cancer; SCLC, small-cell lung cancer; RNAi, RNA interference; siRNA, small interfering RNA; TRAIL, tumor necrosis factor (TNF)-related apoptosis-inducing ligand; CAFs, cancer-associated fibroblasts.

**Reference**

1. Fang JH, Lai YH, Chiu TL *et al*. Magnetic core-shell nanocapsules with dual-targeting capabilities and co-delivery of multiple drugs to treat brain gliomas. Adv Healthc Mater. 2014, 3(8):1250-1260.

2. Jiang T, Sun W, Zhu Q *et al*. Furin-mediated sequential delivery of anticancer cytokine and small-molecule drug shuttled by graphene. Adv Mater. 2015, 27(6):1021-1028.

3. Wang L, Shi J, Zhang H *et al*. Synergistic anticancer effect of RNAi and photothermal therapy mediated by functionalized single-walled carbon nanotubes. Biomaterials. 2013, 34(1):262-274.

4. Xiao Z, Ji C, Shi J *et al*. DNA self-assembly of targeted near-infrared-responsive gold nanoparticles for cancer thermo-chemotherapy. Angew Chem Int Ed Engl. 2012, 51(47):11853-11857.

5. Duan X, Xiao J, Yin Q *et al*. Smart pH-sensitive and temporal-controlled polymeric micelles for effective combination therapy of doxorubicin and disulfiram. ACS Nano. 2013, 7(7):5858-5869.

6. Tardi PG, Dos Santos N, Harasym TO *et al*. Drug ratio-dependent antitumor activity of irinotecan and cisplatin combinations in vitro and in vivo. Mol Cancer Ther. 2009, 8(8):2266-2275.

7. Sengupta S, Eavarone D, Capila I *et al*. Temporal targeting of tumour cells and neovasculature with a nanoscale delivery system. Nature. 2005, 436(7050):568-572.

8. Han L, Huang R, Li J *et al*. Plasmid pORF-hTRAIL and doxorubicin co-delivery targeting to tumor using peptide-conjugated polyamidoamine dendrimer. Biomaterials. 2011, 32(4):1242-1252.
